# The toxicity profile and temporal dynamics of dual PD-1/CTLA-4 immune checkpoint blockade: a real-world pharmacovigilance study using the FAERS database

**DOI:** 10.3389/fimmu.2026.1868547

**Published:** 2026-07-09

**Authors:** Yana Yang, Suting Song, Chunbo Fan, Qian Luo, Chunyu Wang, Yan Luo

**Affiliations:** 1 Health Management Center, The First Affiliated Hospital of Chongqing Medical University, Chongqing, China; 2 Radiation Oncology Center, Chongqing University Cancer Hospital, Chongqing, China

**Keywords:** adverse events, combination therapy, drug synergism, immune checkpoint inhibitors, pharmacoepidemiology

## Abstract

**Background:**

Combination therapy with PD-1 and CTLA-4 inhibitors improves survival in advanced cancers but is associated with heightened toxicity. Whether this represents additive toxicity from each drug or a distinct synergistic profile remains unclear. This study utilized the FDA Adverse Event Reporting System (FAERS) to systematically characterize the toxicity landscape of dual PD-1/CTLA-4 blockade.

**Methods:**

We analyzed FAERS database (2004-2024) for nivolumab, ipilimumab, and their combination. Disproportionality analysis was performed using Reporting Odds Ratios (RORs) and the Ω shrinkage measure model to detect drug-drug interactions signals. To explore potential synergistic signals, we used two exploratory criteria: 1) Emergent signals: adverse events (AEs) with a significant reporting association only for the combination; 2) Supra−additive reporting signals: combination ROR at least 50% higher than the higher monotherapy ROR.

**Results:**

We identified 15, 252 combination therapy reports. Disproportionality analysis revealed 23 emergent preferred terms (PTs) unique to the combination, including immune-related AEs like cardiotoxicity (ROR = 1.89). Strong supra-additive reporting signals were observed for immune-mediated hepatitis (ROR = 205.80) and endocrine toxicity (ROR = 557.95). The Ω shrinkage measure model detected statistical interaction signals for cytokine release syndrome (Ω=2.35) and immune-mediated dermatitis (Ω=2.48). Among cases with evaluable onset dates, the majority of severe AEs were reported within the first 90 days after treatment initiation, with descriptive differences observed across cancer types. Integrated analysis revealed hepatobiliary disorders were the most frequently reported AEs (28.1% of 675 cases), followed by endocrine disorders (14.2%).

**Conclusions:**

Our findings are consistent with the hypothesis that dual PD-1/CTLA-4 blockade may be associated with a toxicity profile involving potential supra-additive reporting associations. These signals include emergent safety events not detected with monotherapies, reporting associations in specific organ systems, and observed temporal patterns. These results are hypothesis-generating and await confirmation in independent studies.

## 1 Introduction

Combination therapy with programmed cell death protein 1 (PD-1) and cytotoxic T-lymphocyte-associated protein 4 (CTLA-4) inhibitors has demonstrated substantial clinical efficacy in several advanced solid malignancies, including melanoma, renal cell carcinoma (RCC), and non-small cell lung cancer (NSCLC) (1–3). Landmark trials have consistently shown superior progression-free survival (PFS) for the combination compared to either agent alone. For instance, at 6 months, the PFS rate was 34% for the combination versus 13% for monotherapy in one pivotal study (2). However, this significant improvement in anti-tumor activity comes at the cost of increased toxicity. Approximately 50% of patients receiving combined PD-1/CTLA-4 blockade experience grade 3 or higher treatment-related AEs, a stark contrast to the 20-25% incidence observed with monotherapies (2, 4). A critical, unresolved question in clinical practice is whether the toxicity profile of the combination represents a simple additive effect of the toxicities associated with each individual drug, or if the interaction may be associated with a distinct AE spectrum suggesting potential synergy.

The unique toxicity profile of combined PD-1/CTLA-4 blockade arises from the distinct mechanisms of action of each pathway in immune regulation. CTLA-4 primarily modulates the initial phase of T-cell activation in secondary lymphoid organs, while PD-1 exerts its main function in peripheral tissues, fine-tuning effector T-cell responses (5). Preclinical models suggest that concurrent blockade can produce synergistic immune activation, characterized by enhanced T-cell proliferative bursts and increased differentiation of CD8+ T cells into potent effectors (6). This synergistic activation provides a a potential mechanistic basis for a qualitatively different toxicity profile, not merely a quantitatively increased one.

Real-world evidence from the FAERS database has become instrumental in post-marketing drug safety assessment. Previous FAERS analyses have confirmed significant safety signals for PD-1/PD-L1 inhibitors, including immune-mediated myocarditis and pneumonitis, whereas CTLA-4 inhibitor monotherapy is strongly associated with severe colitis (7, 8). Despite these insights, the large majority of pharmacovigilance studies have focused on monotherapies. Consequently, while the major immune-related toxicities of the PD-1/CTLA-4 combination, such as colitis, hepatitis, pneumonitis, and endocrinopathies, have been well characterized in landmark clinical trials (e.g., CheckMate 067, 214, 227) and some previous FAERS analyses (9, 10), systematic pharmacovigilance studies that specifically quantify potential drug-drug interaction signals and characterize temporal patterns for this combination remain relatively limited. The recent development of novel bispecific antibodies (e.g., QL1706, AK104), engineered for reduced systemic toxicity via mechanisms like shortened CTLA-4-binding arm half-life or enhanced tumor tissue retention, further underscores the need to fully characterize the toxicity of conventional combination therapy (11, 12).

Therefore, the novelty of the present work lies not in rediscovering known adverse events, but in three specific methodological and analytical aspects that have not been systematically applied to this combination in a real−world FAERS analysis. First, we employ the Ω shrinkage measure model, which is specifically designed to detect drug-drug interaction signals beyond additive effects. Second, we operationalize a two-criterion framework (“emergent signals” and “supra-additive signals”) to distinguish combination-specific reporting patterns from those expected by additivity, a conceptual distinction rarely made in previous pharmacovigilance literature. Third, we provide exploratory analyses of temporal dynamics and dose-severity relationships stratified by cancer type, which have not been systematically examined for this combination in a real-world spontaneous reporting database. To explore whether the toxicity profile of dual PD-1/CTLA-4 blockade may reflect additive or potential synergistic signals, we conducted a comprehensive pharmacovigilance study using the FAERS database. Our analysis specifically employed disproportionality analysis using ROR combined with the Ω shrinkage measure model to detect and quantify potential drug-drug interactions signals across the hierarchy of medical terminology, from PTs to system organ classes (SOCs).

## 2 Materials and methods

### 2.1 Data source and processing

**Figure 1:**
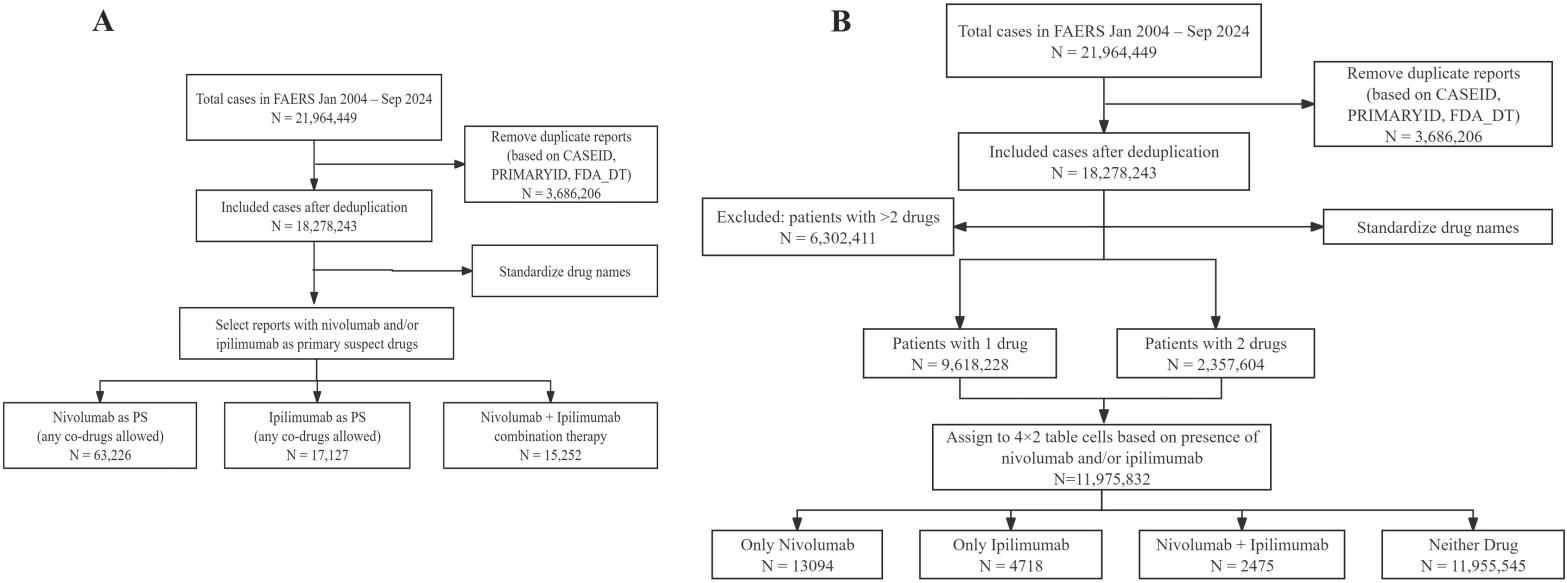
The flow diagram of screening reports from the FAERS database. **(A)** Screening process for monotherapy and combination therapy reports. **(B)** Analytical workflow for the Ω shrinkage measure model.

We conducted a pharmacovigilance study leveraging data from the FAERS (https://open.fda.gov/data/faers/), covering reports from the first quarter of 2004 through the third quarter of 2024 (**Figure 1A**). Data cleaning followed FDA recommendations: duplicate reports were eliminated by retaining the most recent entry based on CASEID and PRIMARYID, with precedence given to later FDA_DT and higher PRIMARYID values for identical cases. For recurring reports pertaining to the same patient, only the most recent record (determined by the “FDA data received to date”) was retained (**Supplementary data 1.1**).

Case reports were identified using a predefined drug name dictionary. For nivolumab, we searched for “nivolumab”, “OPDIVO”, “Nivolumab BMS”, and “OPDYTA”; for ipilimumab, we searched for “ipilimumab”, “Yervoy”, “Ipilimumab recombinant”, “Ipilimumabum”, “MDX 010”, “MDX 101”, “BMS 734016”, and “IBI 310”. Only reports in which an ICI was listed as the “primary suspect” (PS) drug were included. For the disproportionality analysis, the single−drug groups were defined pragmatically to reflect real−world clinical use. The nivolumab group included all reports where nivolumab was listed as PS, regardless of whether ipilimumab or other drugs were also present. The ipilimumab group was defined analogously, including all reports where ipilimumab was listed as PS, irrespective of concomitant medications. The combination group included reports containing both nivolumab and ipilimumab (in any drug role), as the goal was to capture all reports in which both drugs were used, irrespective of which drug was considered the primary suspect.

Key variables obtained from the reports included age, gender, drug names, reporting year, country, and event dates. The onset time of AEs was computed as the interval between therapy initiation (START_DT) and event onset (EVENT_DT). Reports containing chronologically implausible dates (where START_DT occurred later than EVENT_DT) or missing START_DT/EVENT_DT information were excluded from the analysis. AEs were systematically categorized using the Medical Dictionary for Regulatory Activities (MedDRA, version 26.1) across multiple hierarchical levels: SOC, High-Level Group Terms (HLGTs), High-Level Terms (HLTs), and PT.

### 2.2 Statistical methods

Disproportionality analysis was performed using the ROR to compare the frequency of a specific AE associated with a target drug against the frequency of the same AE associated with all other drugs in the FAERS database (13). A standard 2×2 contingency table was constructed for each drug−AE pair as follows:

- number of reports containing the target drug and the target AE;
- number of reports containing the target drug but with other AEs (excluding the target AE);
- number of reports containing other drugs (all drugs in FAERS excluding the target drug) and the target AE;
- number of reports containing other drugs with other AEs.

The formula for calculating ROR is as Equation 1.

(1) $$\text{ROR}=\frac{\left(\text{a}/\text{c}\right)}{\left(\text{b}/\text{d}\right)}=\frac{\text{ad}}{\text{bc}}$$

Signal thresholds were defined as: a lower limit of the 95% CI for ROR (ROR_025_) > 1 with at least 3 reports. The formulas for calculating the ROR, Proportional Reporting Ratio (PRR), Bayesian Confidence Propagation Neural Network (BCPNN), and Multi-item Gamma Poisson Shrinker (MGPS), along with the methods for computing their confidence intervals (CIs) and thresholds, are provided in the supplementary files (**Supplementary data 1.2**).

Additionally, the Ω shrinkage measure model was employed to screen for AE signals associated with the combination of nivolumab and ipilimumab (14, 15). The Ω method estimates drug-drug interactions by comparing the observed frequency of co-reporting for two drugs against the frequency expected if the two drugs acted independently. To make this calculation valid, the exposure categories must be mutually exclusive. Therefore, we used a 4×2 contingency table with the following four groups:

reports containing Nivolumab only (no Ipilimumab);
reports containing Ipilimumab only (no Nivolumab);
reports containing both drugs;
reports containing neither.

The schematic of the data screening process was presented in **Figure 1B**. The Ω statistic is computed using the Equation 2.

(2) $$\Omega =\log_{2}\frac{{\text{n}}_{111}+0.5}{{\text{E}}_{111}+0.5}$$

Here, n_111_ represents the reported number of AEs targeted by the combination of two drugs, and E_111_ denotes the expected value of AEs targeted by the combination of two drugs. The constants 0.5 are added to mitigate small-count variability. A signal is considered significant when the lower bound of the 95% confidence interval for Ω (Ω_025_) exceeds zero, indicating a statistically significant excess in reporting compared to the expected baseline.

The primary data management and all statistical analyses, including descriptive statistics and disproportionality analysis, were conducted in SAS version 9.4 (SAS Institute Inc., Cary, NC, United States). Data visualization was performed using specialized software: time-to-onset analyses were plotted with GraphPad Prism 10.0; Sankey diagram, ridge plots, and box plots were generated using R software (version 4.4.2); and heatmaps were created in Microsoft Excel 2021. This study is designed for safety signal detection using spontaneous reporting data. It cannot estimate incidence, establish causality, or confirm biological synergy. All findings should be interpreted as hypothesis-generating.

### 2.3 Defining potential synergistic signals

Primary evidence for drug−drug interaction: Ω shrinkage model.

The primary method for detecting potential drug−drug interaction was the Ω shrinkage measure model, which is specifically designed to compare observed versus expected co−reporting of two drugs with an AE. A significant Ω signal (Ω_025_ > 0) indicates that the observed number of reports for the combination and a specific AE exceeds the expected number under additivity of the two drugs.

We further defined two exploratory categories to identify AEs that may have a disproportionate reporting association with the combination therapy beyond what would be expected from monotherapies. We emphasize that these definitions are heuristic and do not imply biological synergy or additive risk on a linear scale.

Emergent signals: AEs that did not show a significant signal with either monotherapy (ROR_025_ ≤ 1) but showed a significant reporting association with the combination therapy (ROR_025_ > 1).

Supra-additive reporting signals: AEs meeting either of the following exploratory criteria: (a) the combination ROR was at least 50% higher than the higher of the two monotherapy RORs; or (b) the combination ROR exceeded the sum of the two monotherapy ROR point estimates (a deliberately conservative heuristic). These criteria are not intended as statistical proof of interaction but as a descriptive filter to highlight AEs where the combination showed a notably stronger reporting association than either single agent alone.

## 3 Results

### 3.1 Basic characteristics

This analysis of the FAERS database identified 21, 964, 449 cases of AE reporting from the first quarter of 2004 to the third quarter of 2024 (**Figure 1A**).After deduplication, from the remaining analytic cohort of 11, 975, 832 reports, we identified 63, 226 reports for the nivolumab (nivolumab as primary suspect), 17, 127 for the ipilimumab (ipilimumab as primary suspect), and 15, 252 for the combination group (reports containing both nivolumab and ipilimumab, in any drug role). These three groups are not mutually exclusive for the ROR analysis, as explained in Methods. A male predominance was observed across all groups (nivolumab: 56.43%; ipilimumab: 54.82%; nivolumab + ipilimumab: 56.33%). Patients aged 65 years or older constituted the largest age subgroup (37.79%, 34.99%, and 35.70%, respectively). The majority of reports originated from the United States (42.87%, 43.75%, 49.44%), with Japan being a notable contributor for ipilimumab (22.52%). Regarding reporter type, physicians submitted the most reports for nivolumab (30.62%) and ipilimumab (33.89%), whereas pharmacists were the primary reporters for the combination regimen (31.91%). The reported indications varied substantially, with malignant melanoma being most common for ipilimumab (30.86%) and the combination (22.45%), while NSCLC was a leading indication for nivolumab (11.87%). Analysis of serious outcomes revealed that the combination therapy was associated with the highest rate of hospitalization (48.80%), exceeding both monotherapies (nivolumab: 39.88%; ipilimumab: 42.64%). Mortality was reported in 25.71% of combination therapy cases, which was higher than for ipilimumab (19.78%) but lower than for nivolumab (29.21%). Life-threatening events were most frequently reported with the combination regimen (7.74%) (**Table 1**; **Supplementary Table 1**).

**Table 1:** Clinical characteristics of patients with various ICI regimens.

Characteristics	Nivolumab	Ipilimumab	Nivolumab+Ipilimumab
Gender			
Female (%)	19327 (30.57)	4921 (28.73)	4948 (32.44)
Male (%)	35681 (56.43)	9389 (54.82)	8592 (56.33)
Not Specified (%)	8218 (13.00)	2817 (16.45)	1712 (11.22)
Age			
<18 (%)	241 (0.38)	26 (0.15)	40 (0.26)
18-44 (%)	3190 (5.05)	1101 (6.43)	1092 (7.16)
45-64 (%)	16777 (26.53)	4443 (25.94)	4766 (31.25)
≥65 (%)	23893 (37.79)	5992 (34.99)	5444 (35.7)
Not Specified (%)	19125 (30.25)	5565 (32.49)	3910 (25.64)
Reporting year			
2004~2016 (%)	7685 (12.15)	6642 (38.68)	1002 (6.57)
2017 (%)	7362 (11.64)	1732 (10.11)	1099 (7.21)
2018 (%)	7910 (12.51)	1382 (8.07)	1572 (10.31)
2019 (%)	9100 (14.39)	1081 (6.31)	2361 (15.48)
2020 (%)	8097 (12.81)	949 (5.54)	2273 (14.90)
2021 (%)	7819 (12.37)	991 (5.79)	2404 (15.76)
2022 (%)	7240 (11.45)	1428 (8.34)	2174 (14.25)
2023 (%)	4468 (7.07)	1370 (8.00)	1330 (8.72)
2024 (%)	3545 (5.61)	1570 (9.17)	1037 (6.80)
Reporter type			
Consumer (%)	15752 (24.91)	4789 (27.96)	2778 (18.21)
Other health-professional (%)	12019 (19.01)	3893 (22.73)	3323 (21.79)
Pharmacist (%)	15930 (25.20)	2589 (15.12)	4867 (31.91)
Physician (%)	19362 (30.62)	5804 (33.89)	4264 (27.96)
Lawyer (%)	32 (0.05)	0 (0%)	8 (0.05)
Not Specified (%)	131 (0.21)	52 (0.30)	12 (0.08)
Reporting countries			
United States of America (%)	27108 (42.87)	7493 (43.75)	7541 (49.44)
Japan (%)	9831 (15.55)	3857 (22.52)	1198 (7.85)
France (%)	5711 (9.03)	897 (5.24)	1188 (7.79)
Germany (%)	3084 (4.88)	598 (3.49)	1165 (7.64)
China (%)	2007 (3.17)	45 (0.26)	123 (0.81)
Canada (%)	1709 (2.70)	159 (0.93)	632 (4.14)
Italy (%)	1654 (2.62)	226 (1.32)	233 (1.53)
Australia (%)	1337 (2.11)	351 (2.05)	425 (2.79)
United Kingdom (%)	1069 (1.69)	405 (2.36)	421 (2.76)
India (%)	1011 (1.60)	5 (0.03)	15 (0.10)
Other countries (%)	8705 (13.77)	3091 (18.05)	2311 (15.15)
Indication			
Malignant melanoma (%)	7596 (12.01)	5286 (30.86)	3424 (22.45)
Non-small cell lung cancer (%)	7504 (11.87)	929 (5.42)	1032 (6.77)
Renal cell carcinoma (%)	4085 (6.46)	676 (3.95)	1319 (8.65)
Lung neoplasm malignant (%)	3602 (5.70)	109 (0.64)	294 (1.93)
Metastatic malignant melanoma (%)	3361 (5.32)	2477 (14.46)	1976 (12.96)
Gastric cancer (%)	3167 (5.01)	30 (0.18)	77 (0.50)
Metastatic renal cell carcinoma (%)	2261 (3.58)	700 (4.09)	743 (4.87)
Non-small cell lung cancer recurrent (%)	1638 (2.59)	695 (4.06)	197 (1.29)
Renal cancer (%)	1215 (1.92)	94 (0.55)	266 (1.74)
Squamous cell carcinoma of head and neck (%)	1010 (1.60)	36 (0.21)	48 (0.31)
Other indications (%)	27787 (43.95)	6095 (35.59)	5876 (38.53)
Outcome			
Life-Threatening (%)	4169 (6.59)	927 (5.41)	1181 (7.74)
Hospitalization - Initial or Prolonged (%)	25212 (39.88)	7303 (42.64)	7443 (48.80)
Disability (%)	1054 (1.67)	281 (1.64)	214 (1.40)
Death (%)	18471 (29.21)	3387 (19.78)	3921 (25.71)
Congenital Anomaly (%)	23 (0.04)	5 (0.03)	6 (0.04)

### 3.2 Distribution of AEs at SOC and PT levels

**Figure 2:**
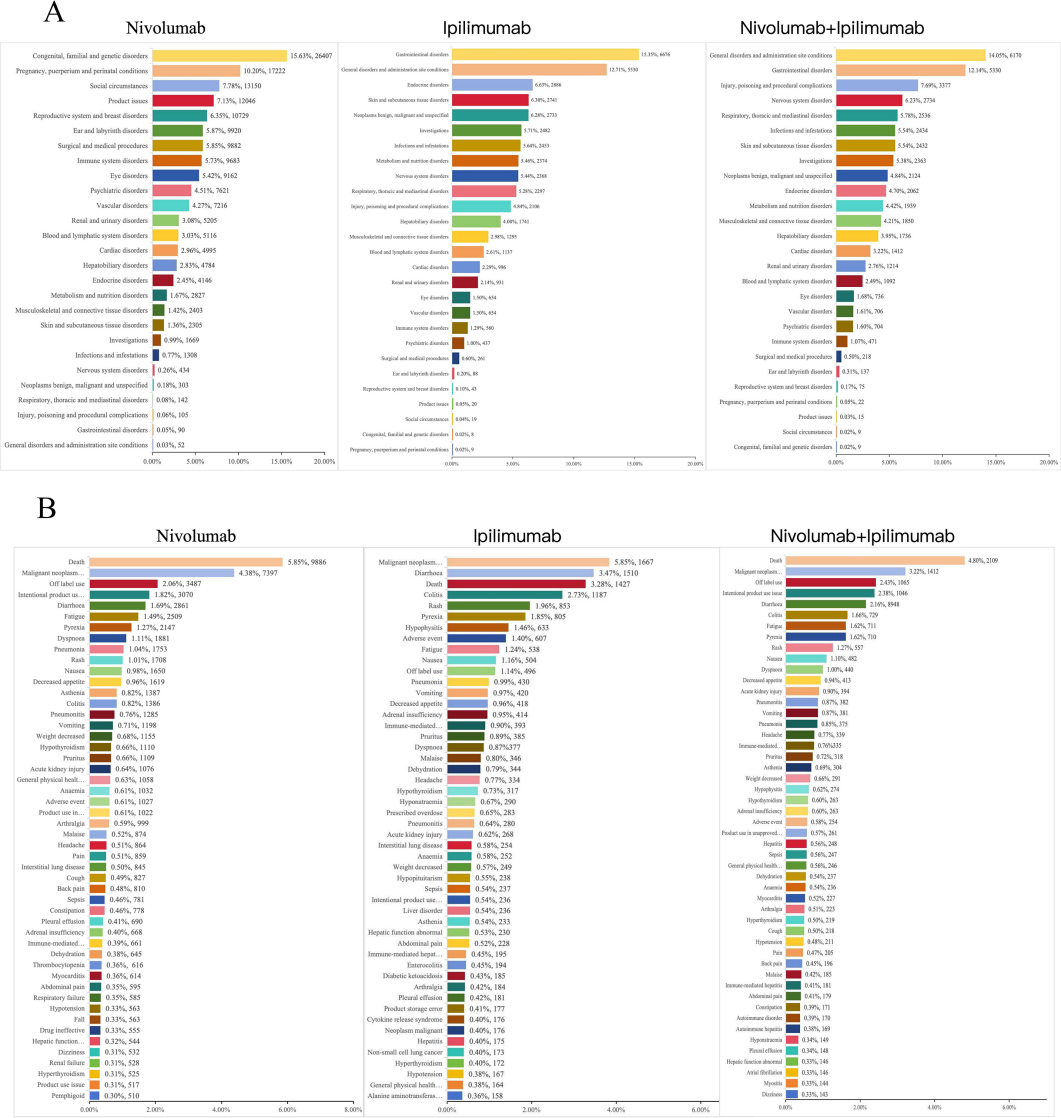
Distribution of AEs for monotherapy and combination therapy regimens. **(A)** Distribution of AEs at SOC levels. **(B)** Distribution of AEs at PT levels.

Based on the analysis of AE profiles at the SOC level, distinct shifts in toxicity patterns were observed between monotherapies and the nivolumab + ipilimumab combination (**Figure 2A**). Compared to nivolumab monotherapy, the combination showed a marked proportional increase in reports for general disorders and administration site conditions (14.05% vs 0.03%), gastrointestinal disorders (12.14% vs 0.05%), and nervous system disorders (6.23% vs 0.26%). A similar pronounced increase was seen versus ipilimumab monotherapy in injury, poisoning and procedural complications (7.69% vs 4.84%) and respiratory, thoracic and mediastinal disorders (5.78% vs 5.28%). Conversely, SOCs that were prominent with single-agent therapy represented a lower proportional burden in the combination regimen. For instance, skin and subcutaneous tissue disorders, a leading SOC for ipilimumab (6.30%), constituted a smaller proportion with combination regimen (5.54%). Similarly, endocrine disorders accounted for a lower proportion in the combination (4.70%) compared to ipilimumab monotherapy (6.63%).

At the more specific PT level, several immune-related AEs showed a pronounced increased reporting proportion in the combination group (**Figure 2B**). Hypophysitis, a well-characterized AE, was reported at a substantially higher proportion with combination (0.62%) compared to ipilimumab monotherapy (0.43%) and was minimal with nivolumab. Similarly, immune-mediated hepatitis (0.41%) and autoimmune hepatitis (0.38%) were notably more prominent with the combination than with either single agent. Myositis (0.33%), while not a top event for either monotherapy, emerged as a significant finding in the combination regimen. Conversely, certain non-specific events represented a lower proportional burden in the combination group. Although malignant neoplasm progression remained a frequent outcome, its proportional reporting was lower in the combination group (3.22%) compared to nivolumab (4.38%) and ipilimumab (3.83%) monotherapies. The combination therapy also altered the landscape of gastrointestinal toxicity. While diarrhoea and colitis were highly prevalent across all regimens, but their relative reporting frequency in the combination group was intermediate.

**Figure 3:**
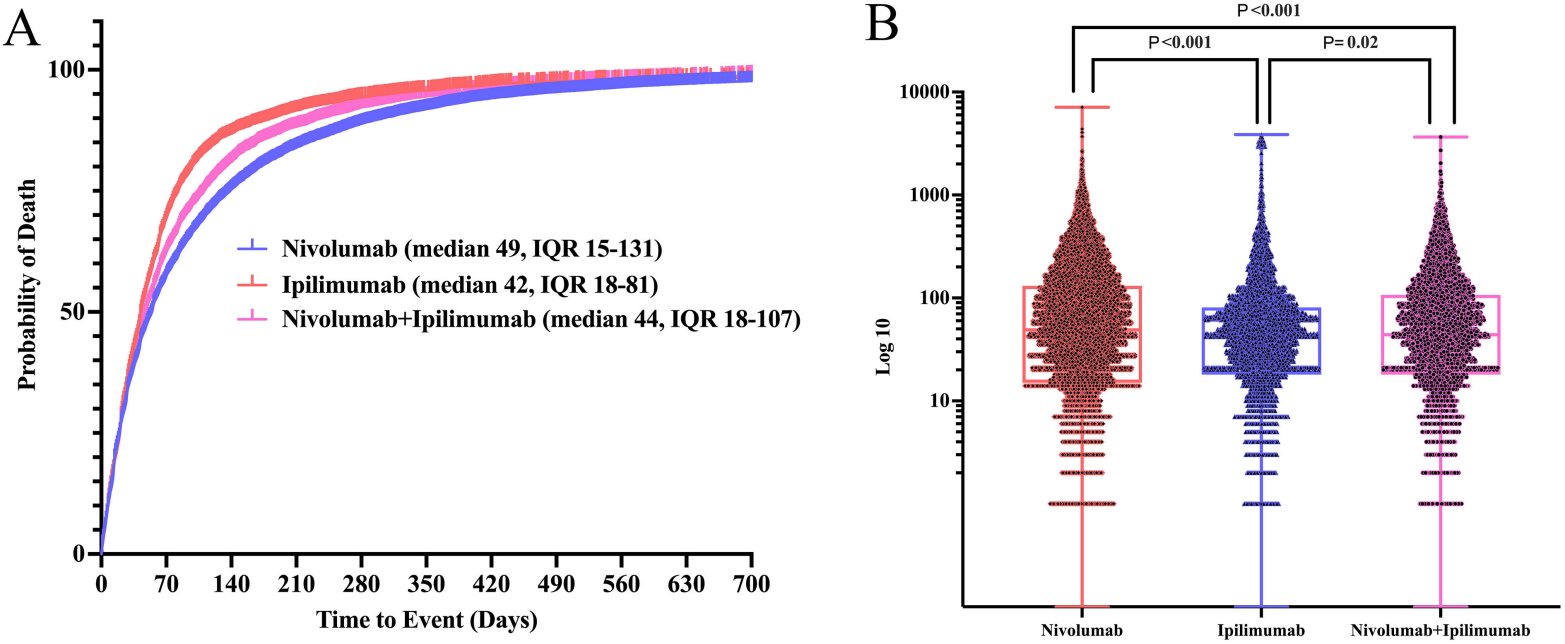
Time-to-onset analysis of ICI-related AEs. **(A)** The cumulative distribution curves of the onset time of ICI-related AEs. **(B)** Comparison of onset time of AEs in various ICI regimens. Statistical tests were conducted using the Kruskal-Wallis’s test.

The time-to-onset analysis included 24, 409 cases for nivolumab, 6, 525 for ipilimumab, and 6, 583 for the nivolumab + ipilimumab combination with available temporal data. A substantial majority of AEs across all regimens occurred within the first three months of treatment initiation. The median time to onset was 49 days (Interquartile Range [IQR]: 15-131) for nivolumab, 42 days (IQR: 18-81) for ipilimumab, and 44 days (IQR: 18-107) for the nivolumab + ipilimumab combination (**Figure 3A**). Statistical comparisons showed a difference in onset time between nivolumab and ipilimumab monotherapies (p< 0.001)). The onset profile of the combination therapy also differed significantly from both nivolumab monotherapy (p< 0.001)) and ipilimumab monotherapy (p=0.02) (**Figure 3B**).

### 3.3 Emergent signals at PT level

**Figure 4:**
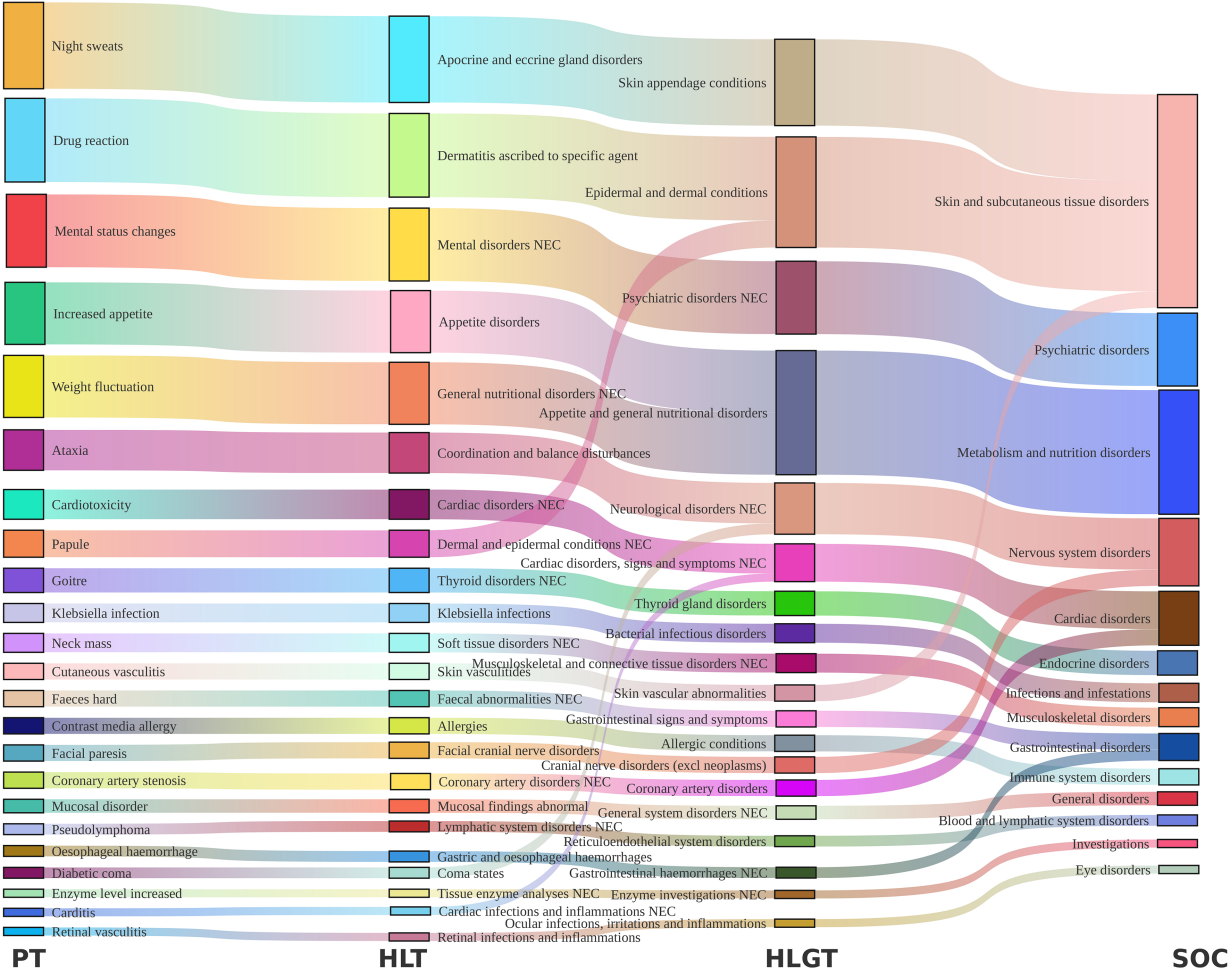
Sankey diagram illustrating the hierarchical relationship of PTs for emergent signals of nivolumab + ipilimumab combination regimen.

Based on the predefined criteria for emergent signals, 23 PTs were identified where the AE did not show a significant signal with either nivolumab or ipilimumab monotherapy but showed a significant reporting association with the combination therapy (**Table 2**). Increasing the minimum case threshold, we repeated the disproportionality analysis using thresholds of n≥5. The emergent signals with ROR > 1 remained significant at both thresholds (**Supplementary Table 2**). We also calculated PRR, BCPNN (IC), and EBGM for the 23 emergent PTs. PRR was fully concordant with ROR. BCPNN detected 14/23, EBGM detected 4/23 using the standard EBGM_05_ ≥ 2 threshold (**Supplementary Table 3**).The most frequently reported events included night sweats (n = 32), drug reaction with eosinophilia and systemic symptoms (n = 31), and mental status changes (n = 27). Immune-related AEs showed notable reporting associations, with carditis showing the highest ROR estimate (ROR = 13.28) despite fewer cases (n = 3). Similarly, pseudolymphoma (n = 4) and contrast media allergy (n = 6) exhibited substantial risk estimates (ROR = 10.87 and 5.81, respectively). Metabolic disorders represented another prominent category, with weight fluctuation (n = 23) and increased appetite (n = 23) among the most common emergent signals. Cardiac disorders manifested through multiple pathways, including cardiotoxicity (n= 11), coronary artery stenosis (n = 6), and carditis (n = 3). The emergent PTs were distributed across multiple SOCs, with the most frequent signals occurring in skin, metabolism, and nervous system disorders. These PTs originate from diverse HLTs, as demonstrated in the Sankey visualization (**Figure 4**). Signals with fewer than 10 reported cases are considered exploratory and are marked with an asterisk (*).

**Table 2:** Emergent signals at PT level for nivolumab + ipilimumab combination regimen.

PT	Nivolumab		Ipilimumab		Nivolumab + Ipilimumab	
	Cases	ROR (ROR_025_-ROR_975_)	Cases	ROR (ROR_025_-ROR_975_)	Cases	ROR (ROR_025_-ROR_975_)
Night sweats	76	0.89 (0.71-1.12)	16	0.73 (0.45-1.19)	32	1.53 (1.08-2.17)
Drug reaction with eosinophilia and systemic symptoms	67	0.88 (0.69-1.12)	8	0.41 (0.2-0.82)	31	1.55 (1.09-2.21)
Mental status changes	88	1.14 (0.92-1.4)	23	1.15 (0.77-1.74)	27	1.6 (1.1-2.34)
Increased appetite	51	1.08 (0.82-1.42)	5	0.41 (0.17-0.98)	23	2.13 (1.41-3.2)
Weight fluctuation	36	1.31 (0.94-1.82)	7	0.99 (0.47-2.07)	23	3.19 (2.12-4.8)
Ataxia	44	1.33 (0.99-1.78)	8	0.93 (0.47-1.87)	15	1.84 (1.11-3.06)
Cardiotoxicity	32	1.39 (0.99-1.97)	2	0.34 (0.08-1.35)	11	1.89 (1.05-3.42)
Papule	21	1.16 (0.75-1.77)	6	1.28 (0.58-2.85)	10	1.96 (1.05-3.64)
Goitre*	17	1.45 (0.9-2.34)	5	1.66 (0.69-3.99)	9	3.24 (1.68-6.23)
Klebsiella infection*	12	0.91 (0.52-1.6)	1	0.29 (0.04-2.09)	7	2.3 (1.09-4.82)
Neck mass*	12	1.57 (0.89-2.78)	3	1.53 (0.49-4.73)	7	3.56 (1.7-7.48)
Cutaneous vasculitis*	17	1.31 (0.82-2.11)	4	1.2 (0.45-3.19)	6	2.65 (1.19-5.9)
Faeces hard*	13	1.28 (0.74-2.21)	2	0.77 (0.19-3.06)	6	2.37 (1.06-5.28)
Contrast media allergy*	8	2 (1-4.01)	1	0.97 (0.14-6.88)	6	5.81 (2.6-12.96)
Facial paresis*	11	1.07 (0.59-1.94)	4	1.52 (0.57-4.05)	6	2.47 (1.11-5.5)
Coronary artery stenosis*	14	0.95 (0.56-1.6)	3	0.79 (0.25-2.45)	6	2.43 (1.09-5.4)
Mucosal disorder*	11	1.65 (0.91-2.98)	1	0.58 (0.08-4.12)	5	3.53 (1.47-8.49)
Pseudolymphoma*	4	2.32 (0.87-6.2)	1	2.24 (0.32-15.97)	4	10.87 (4.06-29.1)
Oesophageal hemorrhage*	6	1.61 (0.72-3.58)	2	2.08 (0.52-8.31)	4	4.48 (1.68-11.97)
Diabetic coma*	7	0.84 (0.4-1.77)	2	0.94 (0.23-3.75)	4	3.18 (1.19-8.47)
Enzyme level increased*	4	1.75 (0.66-4.68)	1	1.7 (0.24-12.07)	3	4.61 (1.48-14.31)
Carditis*	3	3.07 (0.99-9.58)	1	3.96 (0.56-28.22)	3	13.28 (4.25-41.47)
Retinal vasculitis*	5	1.66 (0.69-3.99)	2	2.57 (0.64-10.29)	3	3.83 (1.23-11.89)

*Signals with case count<10 are exploratory.

### 3.4 Supra-additive reporting signals

**Figure 5:**
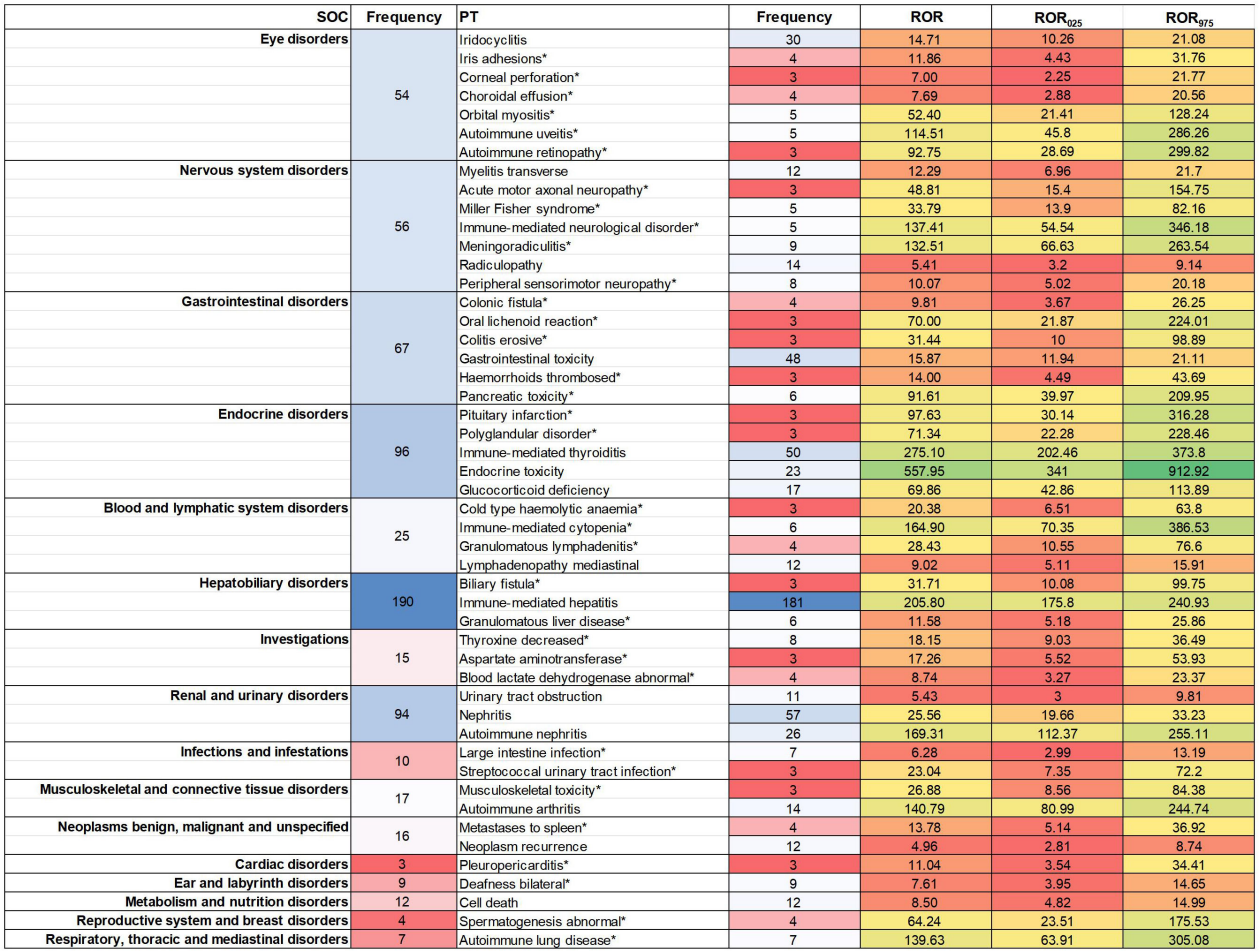
Heatmap displaying supra-additive signals for nivolumab + ipilimumab combination regimen. Note: Signals based on fewer than 10 reports be interpreted as exploratory. * indicated signals with fewer than 10 reported cases.

Supra-additive reporting signals were identified across multiple SOCs (**Figure 5**; **Supplementary Table 4**). The nivolumab + ipilimumab combination therapy showed prominent reporting signals in hepatobiliary disorders (n = 190), primarily driven by immune-mediated hepatitis (ROR = 205.80). Endocrine disorders (n = 96) showed the highest ROR estimates, particularly endocrine toxicity (ROR = 557.95) and immune-mediated thyroiditis (ROR = 275.10). Notable autoimmune manifestations included autoimmune nephritis (ROR = 169.31), autoimmune arthritis (ROR = 140.79), and autoimmune lung disease (ROR = 139.63). Neurological disorders featured immune-mediated neurological disorder (ROR = 137.41) and meningoradiculitis (ROR = 132.51). Ocular disorders revealed several rare but strongly associated events, such as autoimmune uveitis (ROR = 114.51) and orbital myositis (ROR = 52.40). The data further identified significant immune-mediated hematological events, particularly immune-mediated cytopenia (ROR = 164.90). Additional significant signals emerged across renal, gastrointestinal, and musculoskeletal systems, collectively suggesting broad-based supra-additive signals spanning multiple physiological domains.

### 3.5 Ω Shrinkage measure model signal

Based on the Ω shrinkage measure analysis of nivolumab and ipilimumab drug interactions, multiple significant signals were detected across various SOCs (**Table 3**; **Supplementary Table 5**). The most frequently detected drug interaction signals emerged in immune system disorders, with cytokine release syndrome (Ω = 2.35, n = 122) and immune-mediated dermatitis (Ω = 2.48, n = 90) showing strong associations. Hepatobiliary disorders showed significant interaction signals for granulomatous liver disease (Ω = 2.9, n = 7) and hepatic cytolysis (Ω = 2.85, n = 38). Endocrine disorders revealed notable interaction patterns, particularly for endocrine toxicity(Ω = 4.55, n = 23) and immune-mediated endocrinopathy (Ω = 3.27, n = 14). Respiratory disorders exhibited strong signals for Pleural disorder (Ω = 2.27, n = 3), while reproductive system disorders showed spermatogenesis abnormal (Ω = 3.02, n = 4) with the highest interaction measure. Neurological conditions demonstrated significant drug interactions for axonal and demyelinating polyneuropathy (Ω = 2.22, n = 2). Ocular disorders featured hypotony of eye (Ω = 2.64, n = 4) and autoimmune uveitis (Ω = 2.17, n = 4) as notable interaction signals. The analysis detected statistical interaction signals across multiple immune-mediated conditions, with particularly strong associations in dermatological, hepatic, endocrine, and immunological disorders.

**Table 3:** Summary of the top signals per Preferred Terms (PT) using shrinkage measure model for nivolumab + ipilimumab combination regimen.

SOC	PT	Frequency	Ω	Ω_025_	Ω_095_
Endocrine disorders	Endocrine toxicity	23	4.55	3.96	5.14
	Immune-mediated endocrinopathy	14	3.27	2.51	4.02
	Pituitary infarction*	3	2.78	1.15	4.41
	Thyroid stimulating hormone deficiency*	3	2.78	1.14	4.41
	Polyglandular disorder*	3	2.74	1.11	4.37
	Thyroiditis acute*	3	2.61	0.98	4.24
	Immune-mediated hyperthyroidism	14	2.53	1.77	3.28
	Primary hypoparathyroidism*	2	2.3	0.3	4.3
	Immune-mediated adrenal insufficiency	45	1.79	1.37	2.21
	Immune-mediated hypophysitis	63	1.51	1.15	1.86
	Immune-mediated thyroiditis	56	1.45	1.07	1.83
Nervous system disorders	Axonal and demyelinating polyneuropath*y	2	2.22	0.22	4.22
	Immune-mediated neurological disorder*	6	2.11	0.95	3.26
	Acute motor-sensory axonal neuropathy*	2	2.08	0.08	4.07
	Bulbar palsy*	2	2.05	0.05	4.05
	Immune-mediated myasthenia gravis	26	1.63	1.08	2.19
Infections and infestations	Encephalitis brain stem*	3	2.66	1.03	4.3
	Epiglottitis*	3	2.23	0.6	3.86
	Mucosal infection*	2	2.14	0.14	4.14
	Cytomegalovirus infection reactivation*	4	2.12	0.71	3.54
	Pyelonephritis acute*	4	2.11	0.7	3.53
	Cytomegalovirus hepatitis*	2	2.09	0.09	4.08
	Pseudomonal sepsis*	3	2.04	0.41	3.68
Gastrointestinal disorders	Eosinophilic gastritis*	3	2.77	1.14	4.4
	Haemorrhoids thrombosed*	3	2.47	0.84	4.1
	Salivary duct inflammation*	2	2.3	0.3	4.3
	Salivary hyposecretion*	4	2.23	0.82	3.64
	Proctitis hemorrhagic*	2	2.23	0.23	4.23
	Oesophageal achalasia*	3	2.16	0.53	3.79
Cardiac disorders	Carditis*	3	2.35	0.72	3.99
	Cardiac sarcoidosis*	2	2.1	0.1	4.1
	Immune-mediated myocarditis	125	1.03	0.77	1.28
Respiratory disorders	Pleural disorder*	3	2.27	0.63	3.9
	Diffuse panbronchiolitis*	2	2.26	0.27	4.26
	Mediastinal hemorrhage*	2	2.19	0.19	4.19
	Paranasal sinus inflammation*	2	2.13	0.13	4.12
	Autoimmune lung disease*	6	2.11	0.95	3.26
Hepatobiliary disorders	Granulomatous liver disease*	7	2.9	1.83	3.97
	Hepatic cytolysis	38	2.85	2.39	3.31
	Subacute hepatic failure*	2	2.18	0.18	4.18
	Immune-mediated hepatic disorder	143	1.67	1.43	1.91
Skin and subcutaneous tissue disorders	Achromotrichia acquired*	7	2.9	1.83	3.97
	Immune-mediated dermatitis	90	2.48	2.18	2.78
Metabolism and nutrition disorders	Steroid diabetes*	4	2.46	1.04	3.87
	Latent autoimmune diabetes in adults*	2	2.12	0.12	4.12
Blood and lymphatic system disorders	Immune-mediated pancytopenia*	3	2.78	1.15	4.41
	Pseudolymphoma*	4	2.6	1.18	4.01
	Immune-mediated cytopenia*	4	2.17	0.75	3.58
	Bone marrow infiltration*	2	2.02	0.02	4.02
Eye disorders	Hypotony of eye*	4	2.64	1.22	4.05
	Autoimmune uveitis*	4	2.17	0.75	3.58
Renal and urinary disorders	Glomerulonephritis rapidly progressive*	9	1.92	0.98	2.86
	Urinary tract obstruction*	8	1.49	0.5	2.49
Musculoskeletal and connective tissue disorders	Musculoskeletal toxicit*y	3	2.62	0.98	4.25
	Immune-mediated myositis	51	1.19	0.79	1.58
Immune system disorders	Cytokine release syndrome	122	2.35	2.09	2.61
Vascular disorders	Shock	19	0.78	0.13	1.43
Reproductive system and breast disorders	Spermatogenesis abnormal*	4	3.02	1.6	4.43
Ear and labyrinth disorders	Vestibular disorder*	4	1.58	0.16	2.99

* Signals with case count<10 are exploratory.

**Figure 6:**
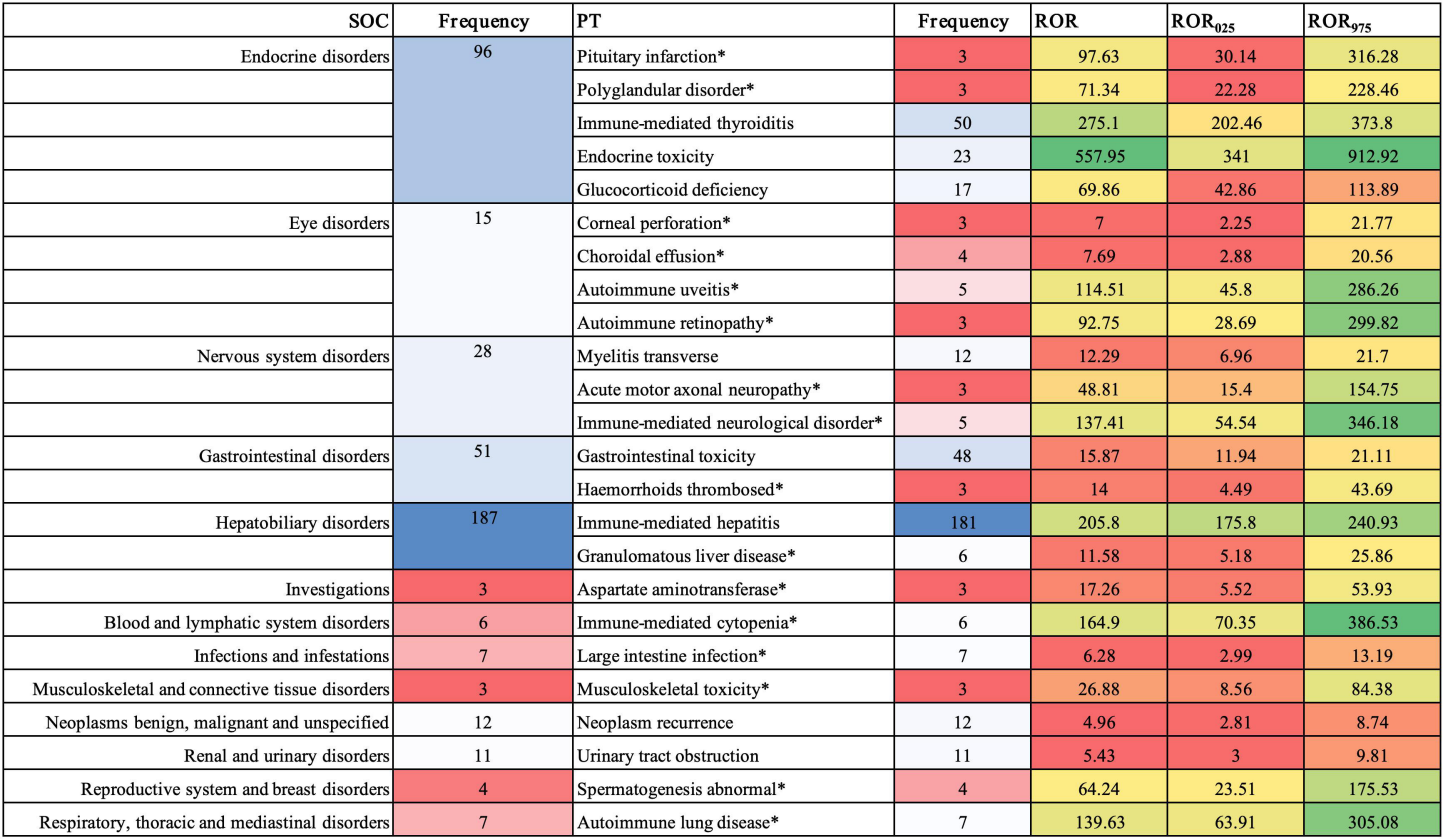
Heatmap displaying the intersection analysis of synergistic signals and Ω shrinkage measure models for the nivolumab + ipilimumab combination regimen. Signals with case count<10 are exploratory. * indicated signals with fewer than 10 reported cases.

The intersection analysis of synergistic signals and Ω shrinkage measure models identified convergent safety signals across multiple SOCs (**Figure 6**; **Supplementary Table 5**). Endocrine disorders showed the most pronounced convergent signals, with endocrine toxicity (ROR = 557.95, n = 23) and immune-mediated thyroiditis (ROR = 275.10, n = 50) showing both strong reporting associations (ROR) and Ω−based interaction signals. Hepatobiliary disorders featured immune-mediated hepatitis (ROR = 205.80, n = 181) as the predominant convergent signal. Immune-mediated conditions displayed strong convergence, including autoimmune lung disease (ROR = 139.63, n = 7), immune-mediated neurological disorder (ROR = 137.41, n = 5), and immune-mediated cytopenia (ROR = 164.90, n = 6). Ocular disorders revealed autoimmune uveitis (ROR = 114.51, n = 5) and autoimmune retinopathy (ROR = 92.75, n = 3) as significant convergent signals. Additional convergent signals emerged across gastrointestinal disorders, nervous system disorders, and reproductive system disorders. Signals with fewer than 10 cases (e.g., spermatogenesis abnormal, autoimmune uveitis) are exploratory and require confirmation.

### 3.6 An integrated analysis of age, time, and dose relationships

All sub−analyses were performed on the nivolumab+ipilimumab combination cohort (N = 15, 252) after applying the Ω shrinkage measure model (N = 2, 457). Because FAERS fields have different completeness, each analysis used a different subset depending on which variables were required. Time−to−onset needed both start and event dates, giving 675 cases. Dose−severity required nivolumab and ipilimumab doses; 371 cases lacked the nivolumab dose and 395 lacked the ipilimumab dose, leaving 271 with complete dose information. The Sankey diagram needed age, gender, cancer type, and severity simultaneously; age was missing in 162 reports and gender in 127, so 499 cases had all four variables. Time−to−severe−event by cancer type was restricted to melanoma (387), renal cell carcinoma (93) and non−small cell lung cancer (44) because each had at least 40 cases with a known severe outcome and onset time, totalling 519. No case was excluded for any reason other than missing data in the required fields (**Supplementary Figure 1**).

**Figure 7:**
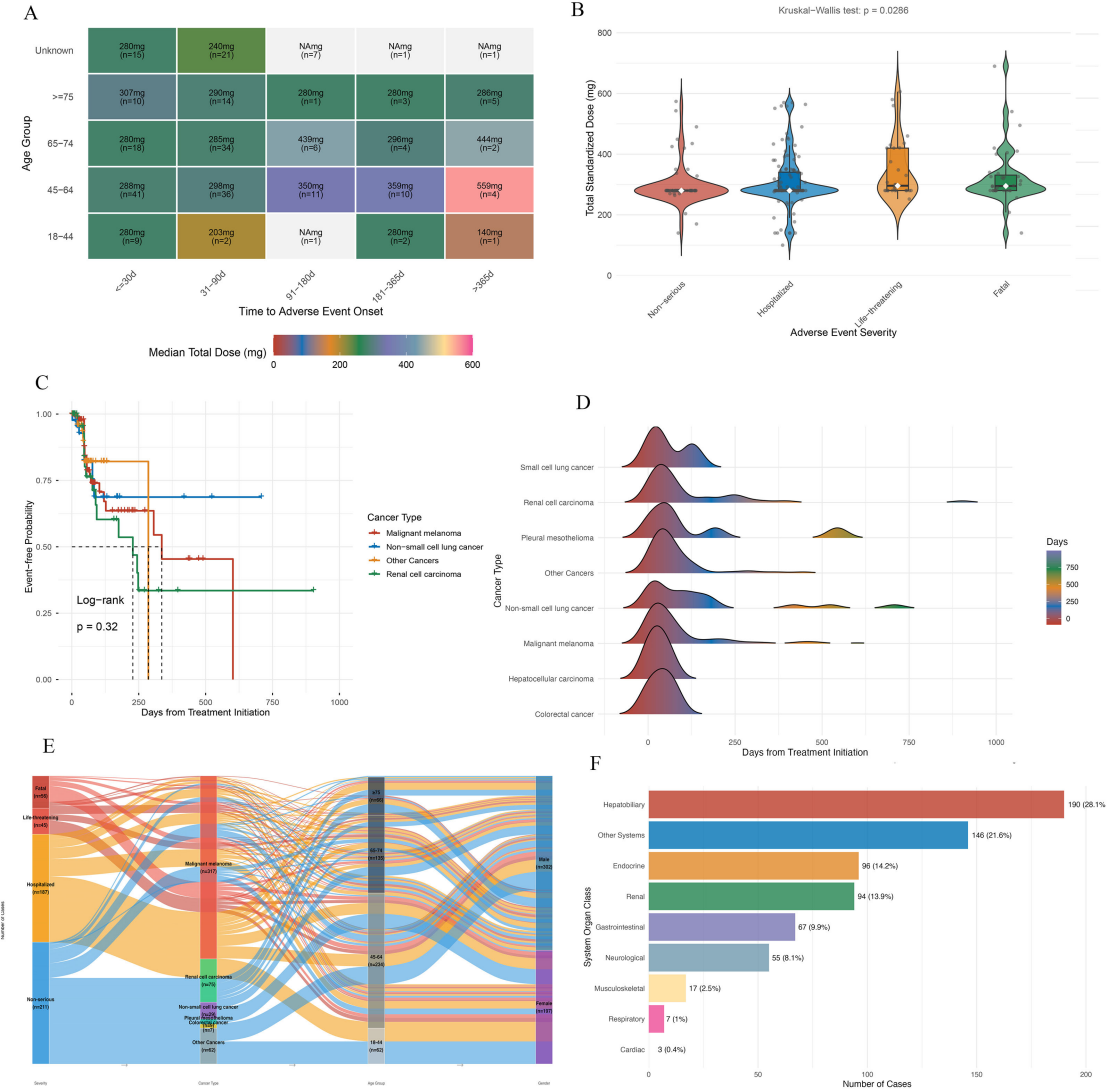
An integrated analysis of age, time, and dose relationships based on Ω shrinkage measure model signals. **(A)** Stratified drug exposure patterns by age and time to onset. FAERS records dose per administration (mg or mg/kg), but not frequency or cumulative cycles. We therefore calculated the summed single−administration dose (nivolumab mg + ipilimumab mg). **(B)** Dose-response relationship in nivolumab + ipilimumab therapy. **(C)** Time to severe AE by cancer types. **(D)** Onset time distributions of AE by cancer types. **(E)**, Multidimensional patient flow through age, cancer type, gender and event severity. **(F)** SOC distribution of AEs.

In the exploratory analysis based on 271 cases with complete dose information, doses recorded as mg/kg were converted to absolute mg using a standard average body weight of 70 kg, which introduces approximation. During the initial 90 days, most age groups showed comparable dose levels (280−307 mg); beyond 90 days, patients aged 45−64 years showed doses up to 559 mg, while those ≥75 years remained at 280−307 mg (Kruskal−Wallis p=0.0895) (**Figure 7A**). Median total dose was 280 mg for non−serious and hospitalized events, and 296 mg for life−threatening and fatal events (p=0.0286) (**Figure 7B**). Given the small subset size, missing dose information, and approximation in unit conversion, this analysis is highly exploratory and does not affect the main conclusions.

A descriptive time-to-severe-event (fatal and life-threatening events) analysis was performed on 519 patients from three cancer types with at least 40 reported cases each (**Figure 7C**). Among melanoma cases (n=383), 55 severe events were reported, with a severe−event−free survival proportion of 85.64% in this reported cohort. Of these severe events, 16 out of 22 (72.73%) with evaluable onset dates occurred within 90 days of treatment initiation. For RCC (n=93), 22 severe events were reported; among those with onset dates, 10 out of 15 (66.67%) occurred within the first 90 days. For NSCLC (n=43), 10 severe events were reported, all of which occurred within 90 days. The log-rank test showed no statistically significant difference in time-to-severe-event distributions among these three cancer types (p=0.32). In the broader onset time analysis of 675 cases, the median time to any AE was 48 days. Melanoma showed the widest reported range (1−600 days). RCC exhibited a bimodal pattern with early and late peaks. NSCLC showed considerable variability in event timing. Small cell lung cancer (SCLC) showed two distinct early peaks. Pleural mesothelioma events clustered within 100 days, and hepatocellular and colorectal cancers showed similarly early−onset patterns (**Figure 7D**).

The Sankey diagram illustrated patient flow through four dimensions in 499 cases (**Figure 7E**). Severe events concentrated in malignant melanoma (n=317), while non-serious events (n=211) distributed more uniformly across cancer types. Malignant melanoma predominated in middle-aged and older adults (45–74 years), whereas RCC occurred more frequently in patients ≥65 years. The 45–64 year age group contributed the largest proportion of cases (n=234), with consistent male predominance across all strata (n=302). SOC distribution analysis of 675 cases identified hepatobiliary disorders as the most common AEs (190 cases, 28.1%), followed by endocrine disorders (96 cases, 14.2%), renal disorders (94 cases, 13.9%), and gastrointestinal events (67 cases, 9.9%). Musculoskeletal (17 cases, 2.5%), respiratory (7 cases, 1.0%), and cardiac disorders (3 cases, 0.4%) were less frequently reported (**Figure 7F**). Comparisons of included versus excluded cases for the onset-time, dose-severity, and cancer-specific analyses showed balanced age and gender distributions. Differences in cancer type and severity reflect the known clinical profile of the combination (**Supplementary Tables 11**–**13**).

## 4 Discussion

Our real-world pharmacovigilance study detects safety signals suggesting that the adverse event profile of combined PD-1/CTLA-4 blockade may not be a simple summation of the individual monotherapy toxicities. Instead, it indentifies a distinct AE pattern characterized by emergent signals, potential supra-additive reporting associations, and Ω shrinkage measure analysis. These hypothesis−generating findings suggest potential areas for further investigation in clinical practice and in the immunobiology of immune-related AEs.

The identification of 23 emergent PT signals exclusive to the combination therapy in this analysis is notable and warrants further investigation.Events such as pseudolymphoma (n=4, ROR = 10.87) and carditis (n=3, ROR = 13.28) showed no significant association with either monotherapy but were detected as signals with the combination, based on small case numbers. This raises the hypothesis that dual checkpoint blockade may unmasks or potentiates novel autoimmune phenomena not typically associated with either pathway alone. The broad distribution of these emergent signals across SOCs like skin, metabolism, and nervous system disorders implies a widespread breakdown of immune tolerance (16, 17). This is consistent with preclinical models demonstrating that concurrent PD-1 and CTLA-4 inhibition induces a qualitatively different T-cell activation state, characterized by enhanced proliferative bursts and diversification of the T-cell receptor repertoire (18, 19). Our findings translate these mechanistic insights into clinical observations, showing that this synergistic immune activation manifests as a broader spectrum of autoimmune-like toxicities.

The potential supra-additive reporting associations observed in hepatobiliary, endocrine, renal and gastrointestinal system disorders suggest specific organ systems that may be at increased risk, pending confirmation. The exceptionally high ROR for immune-mediated hepatitis (ROR = 205.80) and endocrine toxicity (ROR = 557.95) with the combination far exceeds what would be expected from additive effects. This finding is strongly supported by evidence from landmark clinical trials. In CheckMate 067, combination therapy demonstrated significantly higher rates of grade 3–4 AEs (59% versus 28% with ipilimumab, and 25% with nivolumab), indicating a synergistic rather than simply additive toxicity profile (20). Notably, this enhanced toxicity led to treatment discontinuation in over one-third (36%) of patients receiving the combination, underscoring the necessity for vigilant monitoring and aggressive management of AEs. Specific toxicities driving this trend included severe colitis (11% versus 5% with ipilimumab, and< 2% with nivolumab) and any-grade hypophysitis (8% versus 4% with ipilimumab, and< 2% with nivolumab). Similarly, CheckMate 214 and CheckMate 227 trials confirmed this pattern in RCC and NSCLC, respectively, with consistent organ-specific toxicity enhancement (1, 3). Mechanistically, this organ-specific vulnerability could be related to tissue-specific antigen expression and local immune environments. For hepatobiliary disorders, dual ICI blockade, enhances antitumor T-cell activity but disrupts immune self-tolerance, leading to hepatobiliary toxicities including hepatocellular injury and cholestasis (21, 22). Additionally, gene expression analyses indicate dysregulation in signaling pathways encompassing biliary inflammation, bile acid metabolic disorders, and impaired hepatocellular drug metabolism (23).

The strong reporting signals for endocrine toxicities, particularly hypophysitis and thyroiditis, are consistent with with the known expression of CTLA-4 in pituitary folliculostellate cells (24) and the abundant expression of thyroid antigens, which makes the gland particularly vulnerable to T-cell-mediated autoimmunity (25). Unlike other immune-related AEs that may respond to immunosuppression, endocrine damage is often irreversible (26). This permanence raises the possibility that combination therapy may trigger rapid and complete destruction of endocrine cell populations, possibly through mechanisms involving direct T-cell cytotoxicity against irreplaceable specialized cells. Similarly, the significant neurological events, including immune-mediated neurological disorder (ROR = 137.41) and meningoradiculitis, may reflect destabilization of neuroimmune-regulatory networks and promotion of autoreactive immune responses (16).

The shrinkage measure model detected statistical interaction signals for specific AEs, including immune−mediated adrenal insufficiency (Ω=1.79) and cytokine release syndrome (Ω=2.35), suggesting a possible supra−additive reporting association. The concordance between this model and our predefined synergistic criteria provides cross-method support for these signals (**Supplementary Tables 6**; **7**). Our integrated analysis of temporal patterns provides crucial insights for clinical management. The observed concentration of severe events within the first 90 days, particularly for NSCLC and RCC patients, raises the hypothesis that this initial period may be a high-risk window for immune dysregulation. This timing coincides with the expected peak of T-cell activation and proliferation following checkpoint inhibition (27, 28). The dose-severity relationship we observed, though not demonstrating a classic linear correlation, raises the hypothesis that higher cumulative dose may be associated with more severe autoreactivity (29). Interestingly, the similar dosing patterns across age groups despite different toxicity profiles suggest that chronological age alone may be insufficient for risk stratification, and that immunologic age (30) or organ reserve (31) may be more relevant factors. However, this analysis is exploratory and limited by the fact that FAERS captures only per-administration dose, not dosing frequency or total cumulative exposure. The observed association between higher per-administration dose and event severity should be interpreted with extreme caution. The observed cancer-type-specific patterns in time-to-severe-event, though not statistically significant (log-rank p=0.32), generate exploratory hypotheses for future research. In this descriptive analysis, melanoma showed a numerically wider temporal distribution of severe events. One hypothesis is that this might relate to better-established immune tolerance mechanisms in a disease with a longer history of immunotherapy exposure, or to tumor-intrinsic factors that modulate systemic immune responses (32). For RCC and NSCLC, severe events in this reported cohort tended to occur earlier. This raises the hypothesis that these tumors may coexist with a pre-existing state of immune dysregulation that could be amplified by dual checkpoint blockade (33). These observations are hypothesis-generating and require confirmation in prospective studies with adequate denominators and sample sizes.

Our study has several limitations that prevent causal or quantitative interpretation. First, FAERS is a spontaneous reporting system; it suffers from underreporting, selective reporting, and cannot provide denominators. Therefore, we cannot estimate incidence, prevalence, or absolute risk. Second, the lack of randomization and potential confounding (e.g., by indication, disease severity, or concomitant treatments) means that our observed associations may not be causal. We performed sensitivity analyses stratified by reporter type and country to assess the impact of these factors, and the major signals remained consistent. However, residual confounding by indication, cancer type, and other unmeasured factors cannot be fully excluded in FAERS−based analyses. Our findings should therefore be interpreted as hypothesis−generating, not as adjusted risk estimates. Third, the Ω shrinkage measure and ROR are signal detection tools, not formal interaction tests on individual−level data. Consequently, our findings are strictly hypothesis−generating. They require independent replication in cohorts with complete exposure and outcome data before clinical application. An additional limitation is the substantial proportion of missing data within FAERS. Notably, dose information was absent in approximately 60% of reports that otherwise satisfied the inclusion criteria. This necessitated subgroup analyses on varying subsets of the cohort, which carries a risk of selection bias should the missingness not be random. Although we have transparently reported the missing-data profile for each analysis, the potential for bias cannot be excluded.

## 5 Conclusion

In conclusion, our analysis detects safety signals suggesting that dual PD-1/CTLA-4 blockade may be associated with a qualitatively different adverse event profile. These signals are hypothesis-generating and do not imply established synergistic or supra-additive immune effects. The signals include emergent adverse events not detected with monotherapies, organ-specific reporting patterns, and distinct temporal dynamics. These hypothesis-generating findings suggest that a more nuanced approach to combination immunotherapy management may be warranted, pending independent validation.

## Data Availability

The original contributions presented in the study are included in the article/**Supplementary Material**. Further inquiries can be directed to the corresponding author.
