# Supplementary material for: The toxicity profile and temporal dynamics of dual PD-1/CTLA-4 immune checkpoint blockade: a real-world pharmacovigilance study using the FAERS database
Source: Front Immunol. 2026 Jul 9;17:1868547. doi: 10.3389/fimmu.2026.1868547 (PMC13391816; doi:10.3389/fimmu.2026.1868547)

Supplementary Material

## Supplementary data

1.1 Data‑mining procedures: This data mining study utilized the FAERS database from the U.S. FDA website (updated quarterly since Q1 2004), downloading the ASCII data packages. Statistical analysis was performed using SAS 9.4. Data cleaning involved deduplication by CASEID, FDA_DT, and PRIMARYID (retaining the latest FDA_DT and the highest PRIMARYID for identical CASEID and FDA_DT), and removing reports listed in the deleted files starting from Q1 2019. Adverse event terms were recoded using MedDRA version 26.1 to obtain the corresponding system organ class (SOC) and preferred term (PT). The disproportionality analysis method (2×2 contingency table) was adopted, with common signal detection algorithms including ROR, PRR, MHRA, BCPNN, and MGPS. For the target drug population, only primary suspect (PS) drugs were considered, and drug names were standardized using the WHO Drug Dictionary (March 2024 version).

1.2 Signal detection methods

Reporting Odds Ratio，ROR

| Formula | Threshhold |
| --- | --- |
| 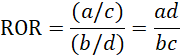  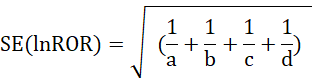  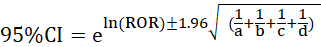 | a ≥ 3 and the lower limit of the 95% confidence interval > 1 |

Proportional Reporting Ratio，PRR

| Formula | Threshhold |
| --- | --- |
| 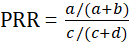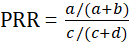  SE(lnPRR)=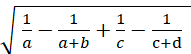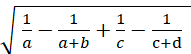  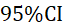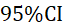=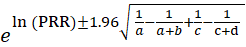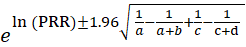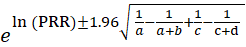  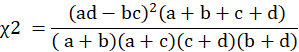 | PRR lower bound of 95% CI method:  a ≥3 and the lower limit of the 95% confidence interval > 1  MHRA combined standard method:  a≥3, PRR value ≥2, and χ2 ≥4 |

Bayesian confidence propagation neural network，BCPNN

| Formula | Threshhold |
| --- | --- |
| IC=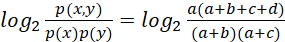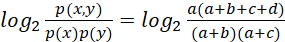  E(IC)=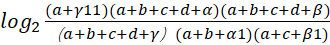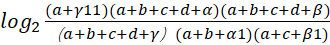  V(IC)=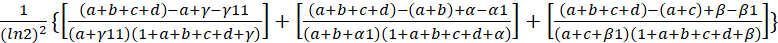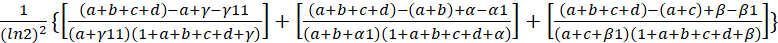  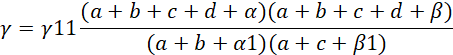  IC-2SD=E(IC)-2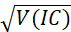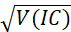  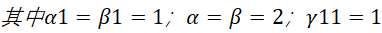 | the lower limit of the 95% credible interval (IC-2SD)＞ 0 |

Muti-item Gamma Poisson Shrinker，MGPS

| Formula | Threshhold |
| --- | --- |
| 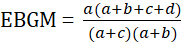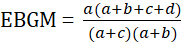  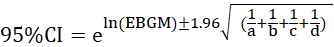 | the lower limit of the 95% confidence interval for EBGM>2 |

# Supplementary Figures and Tables

## Supplementary Tables

Table 1 Detailed clinical characteristics of patients with various ICI regimens

| **Characteristics** | **Nivolumab** | **Ipilimumab** | **Nivolumab+Ipilimumab** |
| --- | --- | --- | --- |
| Gneder |  |  |  |
| Female(%) | 19327(30.57) | 4921(28.73) | 4948(32.44) |
| Male(%) | 35681(56.43) | 9389(54.82) | 8592(56.33) |
| Not Specified(%) | 8218(13.00) | 2817(16.45) | 1712(11.22) |
| Age |  |  |  |
| <18(%) | 241( 0.38) | 26( 0.15) | 40( 0.26) |
| 18-44(%) | 3190( 5.05) | 1101( 6.43) | 1092( 7.16) |
| 45-64(%) | 16777(26.53) | 4443(25.94) | 4766(31.25) |
| ≥65(%) | 23893(37.79) | 5992(34.99) | 5444(35.7) |
| Not Specified(%) | 19125(30.25) | 5565(32.49) | 3910(25.64) |
| Reporting year |  |  |  |
| ~2013(%) | 12( 0.02) | 2560(14.95) | 11( 0.07) |
| 2014(%) | 69( 0.11) | 1349( 7.88) | 68( 0.45) |
| 2015(%) | 1905( 3.01) | 1376( 8.03) | 410( 2.69) |
| 2016(%) | 5699( 9.01) | 1339( 7.82) | 513( 3.36) |
| 2017(%) | 7362(11.64) | 1732(10.11) | 1099( 7.21) |
| 2018(%) | 7910(12.51) | 1382( 8.07) | 1572(10.31) |
| 2019(%) | 9100(14.39) | 1081( 6.31) | 2361(15.48) |
| 2020(%) | 8097(12.81) | 949( 5.54) | 2273(14.90) |
| 2021(%) | 7819(12.37) | 991( 5.79) | 2404(15.76) |
| 2022(%) | 7240(11.45) | 1428( 8.34) | 2174(14.25) |
| 2023(%) | 4468( 7.07) | 1370( 8.00) | 1330( 8.72) |
| 2024(%) | 3545( 5.61) | 1570( 9.17) | 1037( 6.80) |
| Reporter type |  |  |  |
| Consumer | 15752(24.91) | 4789(27.96) | 2778(18.21) |
| Lawyer | 32( 0.05) | 0(0%) | 8( 0.05) |
| Not Specified(%) | 131( 0.21) | 52( 0.30) | 12( 0.08) |
| Other health-professional | 12019(19.01) | 3893(22.73) | 3323(21.79) |
| Pharmacist | 15930(25.20) | 2589(15.12) | 4867(31.91) |
| Physician | 19362(30.62) | 5804(33.89) | 4264(27.96) |
| Reporting countries |  |  |  |
| United States of America(%) | 27108(42.87) | 7493(43.75) | 7541(49.44) |
| Japan(%) | 9831(15.55) | 3857(22.52) | 1198( 7.85) |
| France(%) | 5711( 9.03) | 897( 5.24) | 1188( 7.79) |
| Germany(%) | 3084( 4.88) | 598( 3.49) | 1165( 7.64) |
| China(%) | 2007( 3.17) | 45( 0.26) | 123( 0.81) |
| Canada(%) | 1709( 2.70) | 159( 0.93) | 632( 4.14) |
| Italy(%) | 1654( 2.62) | 226( 1.32) | 233( 1.53) |
| Australia(%) | 1337( 2.11) | 351( 2.05) | 425( 2.79) |
| United Kiongdom(%) | 1069( 1.69) | 405( 2.36) | 421( 2.76) |
| India(%) | 1011( 1.60) | 5( 0.03) | 15( 0.10) |
| Indication |  |  |  |
| Malignant melanoma(%) | 7596(12.01) | 5286(30.86) | 3424(22.45) |
| Non-small cell lung cancer(%) | 7504(11.87) | 929( 5.42) | 1032( 6.77) |
| Renal cell carcinoma(%) | 4085( 6.46) | 676( 3.95) | 1319( 8.65) |
| Lung neoplasm malignant(%) | 3602( 5.70) | 109( 0.64) | 294( 1.93) |
| Metastatic malignant melanoma(%) | 3361( 5.32) | 2477(14.46) | 1976(12.96) |
| Gastric cancer(%) | 3167( 5.01) | 30( 0.18) | 77( 0.50) |
| Metastatic renal cell carcinoma(%) | 2261( 3.58) | 700( 4.09) | 743( 4.87) |
| Non-small cell lung cancer recurrent(%) | 1638( 2.59) | 695( 4.06) | 197( 1.29) |
| Renal cancer(%) | 1215( 1.92) | 94( 0.55) | 266( 1.74) |
| Squamous cell carcinoma of head and neck(%) | 1010( 1.60) | 36( 0.21) | 48( 0.31) |
| Outcome |  |  |  |
| Life-Threatening(%) | 4169( 6.59) | 927( 5.41) | 1181( 7.74) |
| Hospitalization - Initial or Prolonged(%) | 25212(39.88) | 7303(42.64) | 7443(48.80) |
| Disability(%) | 1054( 1.67) | 281( 1.64) | 214( 1.40) |
| Death(%) | 18471(29.21) | 3387(19.78) | 3921(25.71) |
| Congenital Anomaly(%) | 23( 0.04) | 5( 0.03) | 6( 0.04) |

Table 2 Disproportionality analysis using thresholds of n≥5

|  | PT | a | b | c | d | N | ROR | ROR_025_ | ROR_975_ | ROR_Signal |
| --- | --- | --- | --- | --- | --- | --- | --- | --- | --- | --- |
| Nivolumab | Night sweats | 76 | 168846 | 27323 | 54140639 | 54336884 | 0.89 | 0.71 | 1.12 | N |
|  | Drug reaction with eosinophilia and systemic symptoms | 67 | 168855 | 24423 | 54143539 | 54336884 | 0.88 | 0.69 | 1.12 | N |
|  | Mental status changes | 88 | 168834 | 24836 | 54143126 | 54336884 | 1.14 | 0.92 | 1.4 | N |
|  | Increased appetite | 51 | 168871 | 15182 | 54152780 | 54336884 | 1.08 | 0.82 | 1.42 | N |
|  | Weight fluctuation | 36 | 168886 | 8807 | 54159155 | 54336884 | 1.31 | 0.94 | 1.82 | N |
|  | Ataxia | 44 | 168878 | 10649 | 54157313 | 54336884 | 1.33 | 0.99 | 1.78 | N |
|  | Papule | 21 | 168901 | 5830 | 54162132 | 54336884 | 1.16 | 0.75 | 1.77 | N |
|  | Goitre | 17 | 168905 | 3750 | 54164212 | 54336884 | 1.45 | 0.9 | 2.34 | N |
|  |  |  |  |  |  |  |  |  |  |  |
| Ipilimumab | Night sweats | 16 | 43483 | 27383 | 54266002 | 54336884 | 0.73 | 0.45 | 1.19 | N |
|  | Drug reaction with eosinophilia and systemic symptoms | 8 | 43491 | 24482 | 54268903 | 54336884 | 0.41 | 0.2 | 0.82 | N |
|  | Mental status changes | 23 | 43476 | 24901 | 54268484 | 54336884 | 1.15 | 0.77 | 1.74 | N |
|  | Increased appetite | 5 | 43494 | 15228 | 54278157 | 54336884 | 0.41 | 0.17 | 0.98 | N |
|  | Weight fluctuation | 7 | 43492 | 8836 | 54284549 | 54336884 | 0.99 | 0.47 | 2.07 | N |
|  | Ataxia | 8 | 43491 | 10685 | 54282700 | 54336884 | 0.93 | 0.47 | 1.87 | N |
|  | Papule | 6 | 43493 | 5845 | 54287540 | 54336884 | 1.28 | 0.58 | 2.85 | N |
|  | Goitre | 5 | 43494 | 3762 | 54289623 | 54336884 | 1.66 | 0.69 | 3.99 | N |
|  |  |  |  |  |  |  |  | 0 | 0 |  |
| Nivolumab+Ipilimumab | Night sweats | 32 | 41856 | 22170 | 44472613 | 44536671 | 1.53 | 1.08 | 2.17 | Y |
|  | Drug reaction with eosinophilia and systemic symptoms | 31 | 41857 | 21218 | 44473565 | 44536671 | 1.55 | 1.09 | 2.21 | Y |
|  | Mental status changes | 27 | 41861 | 17910 | 44476873 | 44536671 | 1.6 | 1.1 | 2.34 | Y |
|  | Increased appetite | 23 | 41865 | 11492 | 44483291 | 44536671 | 2.13 | 1.41 | 3.2 | Y |
|  | Weight fluctuation | 23 | 41865 | 7665 | 44487118 | 44536671 | 3.19 | 2.12 | 4.8 | Y |
|  | Ataxia | 15 | 41873 | 8639 | 44486144 | 44536671 | 1.84 | 1.11 | 3.06 | Y |
|  | Papule | 10 | 41878 | 5424 | 44489359 | 44536671 | 1.96 | 1.05 | 3.64 | Y |
|  | Goitre | 9 | 41879 | 2955 | 44491828 | 44536671 | 3.24 | 1.68 | 6.23 | Y |

Table 3 Alternative signal detection methods using PRR , BCPNN and EBGM

|  | PT | a | ROR | ROR_025_ | ROR_975_ | Signal | IC | IC_025_ | IC_975_ | Signal | PRR | PRR_025_ | PRR_975_ | Signal | EBGM | EBGM_05_ | EBGM_95_ | Signal |
| --- | --- | --- | --- | --- | --- | --- | --- | --- | --- | --- | --- | --- | --- | --- | --- | --- | --- | --- |
| Nivolumab | Night sweats | 76 | 0.89 | 0.71 | 1.12 | N | -0.16 | -0.49 | -0.49 | N | 0.89 | 0.71 | 1.12 | N | 0.89 | 0.71 | 1.12 | N |
|  | Drug reaction with eosinophilia and systemic symptoms | 67 | 0.88 | 0.69 | 1.12 | N | -0.18 | -0.53 | -0.53 | N | 0.88 | 0.69 | 1.12 | N | 0.88 | 0.69 | 1.12 | N |
|  | Mental status changes | 88 | 1.14 | 0.92 | 1.4 | N | 0.18 | -0.13 | -0.13 | N | 1.14 | 0.92 | 1.4 | N | 1.14 | 0.92 | 1.4 | N |
|  | Increased appetite | 51 | 1.08 | 0.82 | 1.42 | N | 0.11 | -0.3 | -0.3 | N | 1.08 | 0.82 | 1.42 | N | 1.08 | 0.82 | 1.42 | N |
|  | Weight fluctuation | 36 | 1.31 | 0.94 | 1.82 | N | 0.39 | -0.1 | -0.1 | N | 1.31 | 0.94 | 1.82 | N | 1.31 | 0.94 | 1.82 | N |
|  | Ataxia | 44 | 1.33 | 0.99 | 1.78 | N | 0.4 | -0.04 | -0.04 | N | 1.32 | 0.99 | 1.78 | N | 1.32 | 0.98 | 1.78 | N |
|  | Cardiotoxicity | 32 | 1.39 | 0.99 | 1.97 | N | 0.48 | -0.04 | -0.04 | N | 1.39 | 0.99 | 1.97 | N | 1.39 | 0.98 | 1.97 | N |
|  | Papule | 21 | 1.16 | 0.75 | 1.77 | N | 0.21 | -0.42 | -0.42 | N | 1.16 | 0.75 | 1.77 | N | 1.15 | 0.75 | 1.77 | N |
|  | Goitre | 17 | 1.45 | 0.9 | 2.34 | N | 0.54 | -0.18 | -0.18 | N | 1.45 | 0.9 | 2.34 | N | 1.45 | 0.9 | 2.34 | N |
|  | Klebsiella infection | 12 | 0.91 | 0.52 | 1.6 | N | -0.14 | -0.93 | -0.93 | N | 0.91 | 0.52 | 1.6 | N | 0.91 | 0.52 | 1.6 | N |
|  | Neck mass | 12 | 1.57 | 0.89 | 2.78 | N | 0.65 | -0.21 | -0.21 | N | 1.57 | 0.89 | 2.78 | N | 1.57 | 0.89 | 2.77 | N |
|  | Cutaneous vasculitis | 17 | 1.31 | 0.82 | 2.11 | N | 0.39 | -0.32 | -0.32 | N | 1.31 | 0.82 | 2.11 | N | 1.31 | 0.81 | 2.11 | N |
|  | Faeces hard | 13 | 1.28 | 0.74 | 2.21 | N | 0.36 | -0.44 | -0.44 | N | 1.28 | 0.74 | 2.21 | N | 1.28 | 0.74 | 2.21 | N |
|  | Contrast media allergy | 8 | 2 | 1 | 4.01 | N | 1 | -0.12 | -0.12 | N | 2 | 1 | 4.01 | N | 1.99 | 1 | 4 | N |
|  | Facial paresis | 11 | 1.07 | 0.59 | 1.94 | N | 0.1 | -0.74 | -0.74 | N | 1.07 | 0.59 | 1.94 | N | 1.07 | 0.59 | 1.94 | N |
|  | Coronary artery stenosis | 14 | 0.95 | 0.56 | 1.6 | N | -0.08 | -0.82 | -0.82 | N | 0.95 | 0.56 | 1.6 | N | 0.95 | 0.56 | 1.6 | N |
|  | Mucosal disorder | 11 | 1.65 | 0.91 | 2.98 | N | 0.72 | -0.19 | -0.19 | N | 1.65 | 0.91 | 2.98 | N | 1.64 | 0.91 | 2.97 | N |
|  | Pseudolymphoma | 4 | 2.32 | 0.87 | 6.2 | N | 1.21 | -0.43 | -0.43 | N | 2.32 | 0.87 | 6.2 | N | 2.31 | 0.86 | 6.18 | N |
|  | Oesophageal haemorrhage | 6 | 1.61 | 0.72 | 3.58 | N | 0.68 | -0.53 | -0.53 | N | 1.61 | 0.72 | 3.58 | N | 1.6 | 0.72 | 3.58 | N |
|  | Diabetic coma | 7 | 0.84 | 0.4 | 1.77 | N | -0.24 | -1.24 | -1.24 | N | 0.84 | 0.4 | 1.77 | N | 0.84 | 0.4 | 1.77 | N |
|  | Enzyme level increased | 4 | 1.75 | 0.66 | 4.68 | N | 0.81 | -0.69 | -0.69 | N | 1.75 | 0.66 | 4.68 | N | 1.75 | 0.65 | 4.67 | N |
|  | Carditis | 3 | 3.07 | 0.99 | 9.58 | N | 1.61 | -0.44 | -0.44 | N | 3.07 | 0.99 | 9.58 | N | 3.05 | 0.98 | 9.52 | N |
|  | Retinal vasculitis | 5 | 1.66 | 0.69 | 3.99 | N | 0.73 | -0.61 | -0.61 | N | 1.66 | 0.69 | 3.99 | N | 1.65 | 0.69 | 3.98 | N |
|  |  |  |  |  |  |  |  |  |  |  |  |  |  |  |  |  |  |  |
| Ipilimumab | Night sweats | 16 | 0.73 | 0.45 | 1.19 | N | -0.46 | -1.13 | -1.13 | N | 0.73 | 0.45 | 1.19 | N | 0.73 | 0.45 | 1.19 | N |
|  | Drug reaction with eosinophilia and systemic symptoms | 8 | 0.41 | 0.2 | 0.82 | N | -1.29 | -2.16 | -2.16 | N | 0.41 | 0.2 | 0.82 | N | 0.41 | 0.2 | 0.82 | N |
|  | Mental status changes | 23 | 1.15 | 0.77 | 1.74 | N | 0.21 | -0.39 | -0.39 | N | 1.15 | 0.77 | 1.74 | N | 1.15 | 0.77 | 1.74 | N |
|  | Increased appetite | 5 | 0.41 | 0.17 | 0.98 | N | -1.29 | -2.32 | -2.32 | N | 0.41 | 0.17 | 0.98 | N | 0.41 | 0.17 | 0.99 | N |
|  | Weight fluctuation | 7 | 0.99 | 0.47 | 2.07 | N | -0.02 | -1.04 | -1.04 | N | 0.99 | 0.47 | 2.07 | N | 0.99 | 0.47 | 2.07 | N |
|  | Ataxia | 8 | 0.93 | 0.47 | 1.87 | N | -0.1 | -1.05 | -1.05 | N | 0.93 | 0.47 | 1.87 | N | 0.93 | 0.47 | 1.87 | N |
|  | Cardiotoxicity | 2 | 0.34 | 0.08 | 1.35 | N | -1.57 | -2.87 | -2.87 | N | 0.34 | 0.08 | 1.35 | N | 0.34 | 0.08 | 1.35 | N |
|  | Papule | 6 | 1.28 | 0.58 | 2.85 | N | 0.36 | -0.79 | -0.79 | N | 1.28 | 0.58 | 2.85 | N | 1.28 | 0.58 | 2.85 | N |
|  | Goitre | 5 | 1.66 | 0.69 | 3.99 | N | 0.73 | -0.6 | -0.6 | N | 1.66 | 0.69 | 3.99 | N | 1.66 | 0.69 | 3.99 | N |
|  | Klebsiella infection | 1 | 0.29 | 0.04 | 2.09 | N | -1.77 | -3.18 | -3.18 | N | 0.29 | 0.04 | 2.09 | N | 0.29 | 0.04 | 2.09 | N |
|  | Neck mass | 3 | 1.53 | 0.49 | 4.73 | N | 0.61 | -1.01 | -1.01 | N | 1.53 | 0.49 | 4.73 | N | 1.53 | 0.49 | 4.73 | N |
|  | Cutaneous vasculitis | 4 | 1.2 | 0.45 | 3.19 | N | 0.26 | -1.09 | -1.09 | N | 1.2 | 0.45 | 3.19 | N | 1.2 | 0.45 | 3.19 | N |
|  | Faeces hard | 2 | 0.77 | 0.19 | 3.06 | N | -0.39 | -1.94 | -1.94 | N | 0.77 | 0.19 | 3.06 | N | 0.77 | 0.19 | 3.06 | N |
|  | Contrast media allergy | 1 | 0.97 | 0.14 | 6.88 | N | -0.05 | -2.07 | -2.07 | N | 0.97 | 0.14 | 6.88 | N | 0.97 | 0.14 | 6.88 | N |
|  | Facial paresis | 4 | 1.52 | 0.57 | 4.05 | N | 0.6 | -0.83 | -0.83 | N | 1.52 | 0.57 | 4.05 | N | 1.52 | 0.57 | 4.04 | N |
|  | Coronary artery stenosis | 3 | 0.79 | 0.25 | 2.45 | N | -0.34 | -1.71 | -1.71 | N | 0.79 | 0.25 | 2.45 | N | 0.79 | 0.25 | 2.45 | N |
|  | Mucosal disorder | 1 | 0.58 | 0.08 | 4.12 | N | -0.78 | -2.49 | -2.49 | N | 0.58 | 0.08 | 4.12 | N | 0.58 | 0.08 | 4.12 | N |
|  | Pseudolymphoma | 1 | 2.24 | 0.32 | 15.97 | N | 1.17 | -1.58 | -1.58 | N | 2.24 | 0.32 | 15.96 | N | 2.24 | 0.32 | 15.95 | N |
|  | Oesophageal haemorrhage | 2 | 2.08 | 0.52 | 8.31 | N | 1.05 | -1.06 | -1.06 | N | 2.08 | 0.52 | 8.31 | N | 2.08 | 0.52 | 8.31 | N |
|  | Diabetic coma | 2 | 0.94 | 0.23 | 3.75 | N | -0.09 | -1.73 | -1.73 | N | 0.94 | 0.23 | 3.75 | N | 0.94 | 0.23 | 3.75 | N |
|  | Enzyme level increased | 1 | 1.7 | 0.24 | 12.07 | N | 0.76 | -1.71 | -1.71 | N | 1.7 | 0.24 | 12.07 | N | 1.7 | 0.24 | 12.07 | N |
|  | Carditis | 1 | 3.96 | 0.56 | 28.22 | N | 1.98 | -1.37 | -1.37 | N | 3.96 | 0.56 | 28.22 | N | 3.95 | 0.56 | 28.15 | N |
|  | Retinal vasculitis | 2 | 2.57 | 0.64 | 10.29 | N | 1.36 | -0.92 | -0.92 | N | 2.57 | 0.64 | 10.29 | N | 2.57 | 0.64 | 10.28 | N |
|  |  |  |  |  |  |  |  |  |  |  |  |  |  |  |  |  |  |  |
| Nivolumab+  Ipilimumab | Night sweats | 32 | 1.53 | 1.08 | 2.17 | Y | 0.62 | 0.09 | 0.09 | Y | 1.53 | 1.08 | 2.17 | Y | 1.53 | 1.08 | 2.17 | N |
|  | Drug reaction with eosinophilia and systemic symptoms | 31 | 1.55 | 1.09 | 2.21 | Y | 0.63 | 0.1 | 0.1 | Y | 1.55 | 1.09 | 2.21 | Y | 1.55 | 1.09 | 2.21 | N |
|  | Mental status changes | 27 | 1.6 | 1.1 | 2.34 | Y | 0.68 | 0.1 | 0.1 | Y | 1.6 | 1.1 | 2.34 | Y | 1.6 | 1.1 | 2.33 | N |
|  | Increased appetite | 23 | 2.13 | 1.41 | 3.2 | Y | 1.09 | 0.43 | 0.43 | Y | 2.13 | 1.41 | 3.2 | Y | 2.12 | 1.41 | 3.2 | N |
|  | Weight fluctuation | 23 | 3.19 | 2.12 | 4.8 | Y | 1.67 | 0.95 | 0.95 | Y | 3.19 | 2.12 | 4.8 | Y | 3.18 | 2.11 | 4.79 | Y |
|  | Ataxia | 15 | 1.84 | 1.11 | 3.06 | Y | 0.88 | 0.09 | 0.09 | Y | 1.84 | 1.11 | 3.06 | Y | 1.84 | 1.11 | 3.06 | N |
|  | Cardiotoxicity | 11 | 1.89 | 1.05 | 3.42 | Y | 0.92 | -0.02 | -0.02 | N | 1.89 | 1.05 | 3.42 | Y | 1.89 | 1.05 | 3.41 | N |
|  | Papule | 10 | 1.96 | 1.05 | 3.64 | Y | 0.97 | -0.02 | -0.02 | N | 1.96 | 1.05 | 3.64 | Y | 1.96 | 1.05 | 3.64 | N |
|  | Goitre | 9 | 3.24 | 1.68 | 6.23 | Y | 1.69 | 0.49 | 0.49 | Y | 3.24 | 1.68 | 6.22 | Y | 3.23 | 1.68 | 6.21 | N |
|  | Klebsiella infection | 7 | 2.3 | 1.09 | 4.82 | Y | 1.2 | -0.04 | -0.04 | N | 2.3 | 1.09 | 4.82 | Y | 2.29 | 1.09 | 4.82 | N |
|  | Neck mass | 7 | 3.56 | 1.7 | 7.48 | Y | 1.83 | 0.41 | 0.41 | Y | 3.56 | 1.7 | 7.48 | Y | 3.55 | 1.69 | 7.47 | N |
|  | Cutaneous vasculitis | 6 | 2.65 | 1.19 | 5.9 | Y | 1.4 | 0.01 | 0.01 | Y | 2.65 | 1.19 | 5.9 | Y | 2.64 | 1.19 | 5.89 | N |
|  | Faeces hard | 6 | 2.37 | 1.06 | 5.28 | Y | 1.24 | -0.11 | -0.11 | N | 2.37 | 1.06 | 5.28 | Y | 2.37 | 1.06 | 5.27 | N |
|  | Contrast media allergy | 6 | 5.81 | 2.6 | 12.96 | Y | 2.53 | 0.69 | 0.69 | Y | 5.81 | 2.6 | 12.96 | Y | 5.78 | 2.59 | 12.9 | Y |
|  | Facial paresis | 6 | 2.47 | 1.11 | 5.5 | Y | 1.3 | -0.07 | -0.07 | N | 2.47 | 1.11 | 5.5 | Y | 2.46 | 1.11 | 5.49 | N |
|  | Coronary artery stenosis | 6 | 2.43 | 1.09 | 5.4 | Y | 1.28 | -0.08 | -0.08 | N | 2.43 | 1.09 | 5.4 | Y | 2.42 | 1.09 | 5.4 | N |
|  | Mucosal disorder | 5 | 3.53 | 1.47 | 8.49 | Y | 1.82 | 0.13 | 0.13 | Y | 3.53 | 1.47 | 8.49 | Y | 3.52 | 1.46 | 8.47 | N |
|  | Pseudolymphoma | 4 | 10.87 | 4.06 | 29.1 | Y | 3.43 | 0.57 | 0.57 | Y | 10.87 | 4.06 | 29.1 | Y | 10.77 | 4.02 | 28.83 | Y |
|  | Oesophageal haemorrhage | 4 | 4.48 | 1.68 | 11.97 | Y | 2.16 | 0.1 | 0.1 | Y | 4.48 | 1.68 | 11.97 | Y | 4.47 | 1.67 | 11.93 | N |
|  | Diabetic coma | 4 | 3.18 | 1.19 | 8.47 | Y | 1.66 | -0.15 | -0.15 | N | 3.18 | 1.19 | 8.47 | Y | 3.17 | 1.19 | 8.46 | N |
|  | Enzyme level increased | 3 | 4.61 | 1.48 | 14.31 | Y | 2.2 | -0.17 | -0.17 | N | 4.61 | 1.48 | 14.31 | Y | 4.59 | 1.48 | 14.27 | N |
|  | Carditis | 3 | 13.28 | 4.25 | 41.47 | Y | 3.71 | 0.25 | 0.25 | Y | 13.28 | 4.25 | 41.46 | Y | 13.13 | 4.2 | 40.99 | Y |
|  | Retinal vasculitis | 3 | 3.83 | 1.23 | 11.89 | Y | 1.93 | -0.28 | -0.28 | N | 3.83 | 1.23 | 11.89 | Y | 3.82 | 1.23 | 11.85 | N |

Table 4 Supra-additive signals for nivolumab + ipilimumab combination regimen

| SOC | Frequency | PT | Frequency | ROR | ROR_025_ | ROR_975_ | IC | IC_025_ | IC_975_ |
| --- | --- | --- | --- | --- | --- | --- | --- | --- | --- |
| Eye disorders | 54 | Iridocyclitis | 30 | 14.71 | 10.26 | 21.08 | 3.86 | 2.82 | 3.86 |
|  |  | Iris adhesions | 4 | 11.86 | 4.43 | 31.76 | 3.56 | 0.6 | 3.2 |
|  |  | Corneal perforation | 3 | 7 | 2.25 | 21.77 | 2.8 | 0.03 | 2.93 |
|  |  | Choroidal effusion | 4 | 7.69 | 2.88 | 20.56 | 2.94 | 0.42 | 3.01 |
|  |  | Orbital myositis | 5 | 52.4 | 21.41 | 128.24 | 5.65 | 1.24 | 3.65 |
|  |  | Autoimmune uveitis | 5 | 114.51 | 45.8 | 286.26 | 6.71 | 1.28 | 3.75 |
|  |  | Autoimmune retinopathy | 3 | 92.75 | 28.69 | 299.82 | 6.43 | 0.44 | 3.46 |
| Nervous system disorders | 56 | Myelitis transverse | 12 | 12.29 | 6.96 | 21.7 | 3.61 | 1.91 | 3.51 |
|  |  | Acute motor axonal neuropathy | 3 | 48.81 | 15.4 | 154.75 | 5.55 | 0.43 | 3.39 |
|  |  | Miller Fisher syndrome | 5 | 33.79 | 13.9 | 82.16 | 5.04 | 1.18 | 3.58 |
|  |  | Immune-mediated neurological disorder | 5 | 137.41 | 54.54 | 346.18 | 6.95 | 1.28 | 3.77 |
|  |  | Meningoradiculitis | 9 | 132.51 | 66.63 | 263.54 | 6.9 | 2.26 | 4.18 |
|  |  | Radiculopathy | 14 | 5.41 | 3.2 | 9.14 | 2.43 | 1.31 | 2.81 |
|  |  | Peripheral sensorimotor neuropathy | 8 | 10.07 | 5.02 | 20.18 | 3.32 | 1.35 | 3.29 |
| Gastrointestinal disorders | 67 | Colonic fistula | 4 | 9.81 | 3.67 | 26.25 | 3.28 | 0.53 | 3.12 |
|  |  | Oral lichenoid reaction | 3 | 70 | 21.87 | 224.01 | 6.05 | 0.44 | 3.43 |
|  |  | Colitis erosive | 3 | 31.44 | 10 | 98.89 | 4.94 | 0.4 | 3.33 |
|  |  | Gastrointestinal toxicity | 48 | 15.87 | 11.94 | 21.11 | 3.97 | 3.18 | 4.01 |
|  |  | Haemorrhoids thrombosed | 3 | 14 | 4.49 | 43.69 | 3.79 | 0.26 | 3.17 |
|  |  | Pancreatic toxicity | 6 | 91.61 | 39.97 | 209.95 | 6.42 | 1.58 | 3.84 |
| Endocrine disorders | 96 | Pituitary infarction | 3 | 97.63 | 30.14 | 316.28 | 6.5 | 0.44 | 3.46 |
|  |  | Polyglandular disorder | 3 | 71.34 | 22.28 | 228.46 | 6.08 | 0.44 | 3.43 |
|  |  | Immune-mediated thyroiditis | 50 | 275.1 | 202.46 | 373.8 | 7.81 | 4.94 | 5.82 |
|  |  | Endocrine toxicity | 23 | 557.95 | 341 | 912.92 | 8.59 | 3.82 | 5.18 |
|  |  | Glucocorticoid deficiency | 17 | 69.86 | 42.86 | 113.89 | 6.05 | 3.14 | 4.54 |
| Blood and lymphatic system disorders | 25 | Cold type haemolytic anaemia | 3 | 20.38 | 6.51 | 63.8 | 4.33 | 0.34 | 3.26 |
|  |  | Immune-mediated cytopenia | 6 | 164.9 | 70.35 | 386.53 | 7.19 | 1.59 | 3.91 |
|  |  | Granulomatous lymphadenitis | 4 | 28.43 | 10.55 | 76.6 | 4.8 | 0.82 | 3.44 |
|  |  | Lymphadenopathy mediastinal | 12 | 9.02 | 5.11 | 15.91 | 3.16 | 1.67 | 3.28 |
| Hepatobiliary disorders | 190 | Biliary fistula | 3 | 31.71 | 10.08 | 99.75 | 4.95 | 0.4 | 3.33 |
|  |  | Immune-mediated hepatitis | 181 | 205.8 | 175.8 | 240.93 | 7.46 | 6.26 | 6.72 |
|  |  | Granulomatous liver disease | 6 | 11.58 | 5.18 | 25.86 | 3.52 | 1.1 | 3.3 |
| Investigations | 15 | Thyroxine decreased | 8 | 18.15 | 9.03 | 36.49 | 4.16 | 1.67 | 3.61 |
|  |  | Aspartate aminotransferase | 3 | 17.26 | 5.52 | 53.93 | 4.09 | 0.31 | 3.22 |
|  |  | Blood lactate dehydrogenase abnormal | 4 | 8.74 | 3.27 | 23.37 | 3.12 | 0.48 | 3.07 |
| Renal and urinary disorders | 94 | Urinary tract obstruction | 11 | 5.43 | 3 | 9.81 | 2.43 | 1.15 | 2.82 |
|  |  | Nephritis | 57 | 25.56 | 19.66 | 33.23 | 4.65 | 3.76 | 4.53 |
|  |  | Autoimmune nephritis | 26 | 169.31 | 112.37 | 255.11 | 7.22 | 3.93 | 5.11 |
| Infections and infestations | 10 | Large intestine infection | 7 | 6.28 | 2.99 | 13.19 | 2.64 | 0.89 | 2.94 |
|  |  | Streptococcal urinary tract infection | 3 | 23.04 | 7.35 | 72.2 | 4.5 | 0.36 | 3.28 |
| Musculoskeletal and connective tissue disorders | 17 | Musculoskeletal toxicity | 3 | 26.88 | 8.56 | 84.38 | 4.72 | 0.38 | 3.31 |
|  |  | Autoimmune arthritis | 14 | 140.79 | 80.99 | 244.74 | 6.98 | 2.97 | 4.54 |
| Neoplasms benign, malignant and unspecified | 16 | Metastases to spleen | 4 | 13.78 | 5.14 | 36.92 | 3.77 | 0.65 | 3.25 |
|  |  | Neoplasm recurrence | 12 | 4.96 | 2.81 | 8.74 | 2.3 | 1.12 | 2.72 |
| Cardiac disorders | 3 | Pleuropericarditis | 3 | 11.04 | 3.54 | 34.41 | 3.45 | 0.2 | 3.1 |
| Ear and labyrinth disorders | 9 | Deafness bilateral | 9 | 7.61 | 3.95 | 14.65 | 2.92 | 1.28 | 3.11 |
| Metabolism and nutrition disorders | 12 | Cell death | 12 | 8.5 | 4.82 | 14.99 | 3.08 | 1.62 | 3.23 |
| Reproductive system and breast disorders | 4 | Spermatogenesis abnormal | 4 | 64.24 | 23.51 | 175.53 | 5.93 | 0.9 | 3.56 |
| Respiratory, thoracic and mediastinal disorders | 7 | Autoimmune lung disease | 7 | 139.63 | 63.91 | 305.08 | 6.97 | 1.84 | 4 |

Table 5 Intersection analysis of synergistic signals and Ω shrinkage measure models for the nivolumab + ipilimumab combination regimen

| SOC | Frequency | PT | Frequency | ROR | ROR_025_ | ROR_975_ | IC | IC_025_ | IC_975_ |
| --- | --- | --- | --- | --- | --- | --- | --- | --- | --- |
| Endocrine disorders | 96 | Pituitary infarction | 3 | 97.63 | 30.14 | 316.28 | 6.5 | 0.44 | 3.46 |
|  |  | Polyglandular disorder | 3 | 71.34 | 22.28 | 228.46 | 6.08 | 0.44 | 3.43 |
|  |  | Immune-mediated thyroiditis | 50 | 275.1 | 202.46 | 373.8 | 7.81 | 4.94 | 5.82 |
|  |  | Endocrine toxicity | 23 | 557.95 | 341 | 912.92 | 8.59 | 3.82 | 5.18 |
|  |  | Glucocorticoid deficiency | 17 | 69.86 | 42.86 | 113.89 | 6.05 | 3.14 | 4.54 |
| Eye disorders | 15 | Corneal perforation | 3 | 7 | 2.25 | 21.77 | 2.8 | 0.03 | 2.93 |
|  |  | Choroidal effusion | 4 | 7.69 | 2.88 | 20.56 | 2.94 | 0.42 | 3.01 |
|  |  | Autoimmune uveitis | 5 | 114.51 | 45.8 | 286.26 | 6.71 | 1.28 | 3.75 |
|  |  | Autoimmune retinopathy | 3 | 92.75 | 28.69 | 299.82 | 6.43 | 0.44 | 3.46 |
| Nervous system disorders | 28 | Myelitis transverse | 12 | 12.29 | 6.96 | 21.7 | 3.61 | 1.91 | 3.51 |
|  |  | Acute motor axonal neuropathy | 3 | 48.81 | 15.4 | 154.75 | 5.55 | 0.43 | 3.39 |
|  |  | Immune-mediated neurological disorder | 5 | 137.41 | 54.54 | 346.18 | 6.95 | 1.28 | 3.77 |
| Gastrointestinal disorders | 51 | Gastrointestinal toxicity | 48 | 15.87 | 11.94 | 21.11 | 3.97 | 3.18 | 4.01 |
|  |  | Haemorrhoids thrombosed | 3 | 14 | 4.49 | 43.69 | 3.79 | 0.26 | 3.17 |
| Hepatobiliary disorders | 187 | Immune-mediated hepatitis | 181 | 205.8 | 175.8 | 240.93 | 7.46 | 6.26 | 6.72 |
|  |  | Granulomatous liver disease | 6 | 11.58 | 5.18 | 25.86 | 3.52 | 1.1 | 3.3 |
| Investigations | 3 | Aspartate aminotransferase | 3 | 17.26 | 5.52 | 53.93 | 4.09 | 0.31 | 3.22 |
| Blood and lymphatic system disorders | 6 | Immune-mediated cytopenia | 6 | 164.9 | 70.35 | 386.53 | 7.19 | 1.59 | 3.91 |
| Infections and infestations | 7 | Large intestine infection | 7 | 6.28 | 2.99 | 13.19 | 2.64 | 0.89 | 2.94 |
| Musculoskeletal and connective tissue disorders | 3 | Musculoskeletal toxicity | 3 | 26.88 | 8.56 | 84.38 | 4.72 | 0.38 | 3.31 |
| Neoplasms benign, malignant and unspecified | 12 | Neoplasm recurrence | 12 | 4.96 | 2.81 | 8.74 | 2.3 | 1.12 | 2.72 |
| Renal and urinary disorders | 11 | Urinary tract obstruction | 11 | 5.43 | 3 | 9.81 | 2.43 | 1.15 | 2.82 |
| Reproductive system and breast disorders | 4 | Spermatogenesis abnormal | 4 | 64.24 | 23.51 | 175.53 | 5.93 | 0.9 | 3.56 |
| Respiratory, thoracic and mediastinal disorders | 7 | Autoimmune lung disease | 7 | 139.63 | 63.91 | 305.08 | 6.97 | 1.84 | 4 |

Table 6 Comparisoon of the Ω shrinkage measure model and an additive model (Thakrar method)

| SOC | PT | Frequency | Additive_Model | Signal | Ω | Ω_025_ | Ω_095_ | Signal |
| --- | --- | --- | --- | --- | --- | --- | --- | --- |
| Blood and lymphatic system disorders | Febrile neutropenia | 32 | 0.000641536 | Y | 0.77 | 0.27 | 1.27 | Y |
| Blood and lymphatic system disorders | Bone marrow infiltration | 2 | 6.70648E-05 | Y | 2.02 | 0.02 | 4.02 | Y |
| Blood and lymphatic system disorders | Pseudolymphoma | 4 | 0.000134483 | Y | 2.6 | 1.18 | 4.01 | Y |
| Blood and lymphatic system disorders | Immune-mediated pancytopenia | 3 | 9.53489E-05 | Y | 2.78 | 1.15 | 4.41 | Y |
| Blood and lymphatic system disorders | Immune-mediated cytopenia | 4 | 0.000111637 | Y | 2.17 | 0.75 | 3.58 | Y |
| Blood and lymphatic system disorders | Hyposplenism | 2 | 6.36302E-05 | Y | 2.3 | 0.3 | 4.3 | Y |
| Blood and lymphatic system disorders | Splenomegaly | 10 | 0.00023306 | Y | 1.01 | 0.12 | 1.91 | Y |
| Cardiac disorders | Acute myocardial infarction | 24 | 0.000396448 | Y | 0.65 | 0.07 | 1.23 | Y |
| Cardiac disorders | Immune-mediated myocarditis | 125 | 0.002021702 | Y | 1.03 | 0.77 | 1.28 | Y |
| Cardiac disorders | Ventricular tachycardia | 13 | 0.000262891 | Y | 0.94 | 0.15 | 1.72 | Y |
| Cardiac disorders | Ventricular arrhythmia | 5 | 0.000155539 | Y | 1.45 | 0.19 | 2.72 | Y |
| Cardiac disorders | Myocarditis | 195 | 0.001918173 | Y | 0.54 | 0.33 | 0.74 | Y |
| Cardiac disorders | Cardiogenic shock | 23 | 0.000583742 | Y | 1.22 | 0.63 | 1.81 | Y |
| Cardiac disorders | Cardiac sarcoidosis | 2 | 6.60376E-05 | Y | 2.1 | 0.1 | 4.1 | Y |
| Cardiac disorders | Carditis | 3 | 0.000100902 | Y | 2.35 | 0.72 | 3.99 | Y |
| Cardiac disorders | Bundle branch block right | 5 | 0.000175624 | Y | 1.42 | 0.16 | 2.69 | Y |
| Cardiac disorders | Sinus tachycardia | 18 | 0.000282302 | Y | 0.95 | 0.28 | 1.61 | Y |
| Ear and labyrinth disorders | Vestibular disorder | 4 | 0.000111966 | Y | 1.58 | 0.16 | 2.99 | Y |
| Endocrine disorders | Pituitary infarction | 3 | 9.5381E-05 | Y | 2.78 | 1.15 | 4.41 | Y |
| Endocrine disorders | Thyroid stimulating hormone deficiency | 3 | 9.54131E-05 | Y | 2.78 | 1.14 | 4.41 | Y |
| Endocrine disorders | Adrenocorticotropic hormone deficiency | 62 | 0.001148841 | Y | 1.25 | 0.89 | 1.61 | Y |
| Endocrine disorders | Polyglandular disorder | 3 | 9.57983E-05 | Y | 2.74 | 1.11 | 4.37 | Y |
| Endocrine disorders | Thyroiditis acute | 3 | 9.74033E-05 | Y | 2.61 | 0.98 | 4.24 | Y |
| Endocrine disorders | Secondary adrenocortical insufficiency | 74 | 0.001317382 | Y | 1.18 | 0.85 | 1.51 | Y |
| Endocrine disorders | Hypoparathyroidism | 6 | 0.000136517 | Y | 1.37 | 0.22 | 2.53 | Y |
| Endocrine disorders | Thyrotoxic crisis | 15 | 0.000236292 | Y | 0.95 | 0.22 | 1.68 | Y |
| Endocrine disorders | Hyperthyroidism | 162 | 0.001515534 | Y | 0.5 | 0.28 | 0.73 | Y |
| Endocrine disorders | Thyroiditis | 122 | 0.001087213 | Y | 0.48 | 0.22 | 0.73 | Y |
| Endocrine disorders | Immune-mediated endocrinopathy | 14 | 0.000412655 | Y | 3.27 | 2.51 | 4.02 | Y |
| Endocrine disorders | Immune-mediated hypophysitis | 63 | 0.001303615 | Y | 1.51 | 1.15 | 1.86 | Y |
| Endocrine disorders | Immune-mediated hypothyroidism | 46 | 0.00062163 | Y | 0.79 | 0.37 | 1.21 | Y |
| Endocrine disorders | Immune-mediated hyperthyroidism | 14 | 0.000381312 | Y | 2.53 | 1.77 | 3.28 | Y |
| Endocrine disorders | Immune-mediated thyroiditis | 56 | 0.001136008 | Y | 1.45 | 1.07 | 1.83 | Y |
| Endocrine disorders | Immune-mediated adrenal insufficiency | 45 | 0.001025091 | Y | 1.79 | 1.37 | 2.21 | Y |
| Endocrine disorders | Endocrine toxicity | 23 | 0.000713363 | Y | 4.55 | 3.96 | 5.14 | Y |
| Endocrine disorders | Glucocorticoid deficiency | 9 | 0.000204901 | Y | 1.65 | 0.7 | 2.59 | Y |
| Endocrine disorders | Primary hypoparathyroidism | 2 | 6.36623E-05 | Y | 2.3 | 0.3 | 4.3 | Y |
| Endocrine disorders | Primary adrenal insufficiency | 6 | 0.000129551 | Y | 1.37 | 0.22 | 2.53 | Y |
| Eye disorders | Corneal perforation | 3 | 8.73328E-05 | Y | 1.8 | 0.17 | 3.44 | Y |
| Eye disorders | Choroidal effusion | 3 | 9.33675E-05 | Y | 1.8 | 0.17 | 3.44 | Y |
| Eye disorders | Immune-mediated uveitis | 34 | 0.000441608 | Y | 0.75 | 0.26 | 1.23 | Y |
| Eye disorders | Hypotony of eye | 4 | 0.000133841 | Y | 2.64 | 1.22 | 4.05 | Y |
| Eye disorders | Autoimmune uveitis | 4 | 0.000111701 | Y | 2.17 | 0.75 | 3.58 | Y |
| Eye disorders | Autoimmune retinopathy | 3 | 7.9629E-05 | Y | 1.8 | 0.17 | 3.44 | Y |
| Gastrointestinal disorders | Gastritis haemorrhagic | 7 | 0.000161247 | Y | 1.31 | 0.25 | 2.38 | Y |
| Gastrointestinal disorders | Proctitis haemorrhagic | 2 | 6.44326E-05 | Y | 2.23 | 0.23 | 4.23 | Y |
| Gastrointestinal disorders | Malignant ascites | 3 | 8.77501E-05 | Y | 1.8 | 0.17 | 3.44 | Y |
| Gastrointestinal disorders | Immune-mediated enterocolitis | 395 | 0.002421413 | Y | 0.32 | 0.17 | 0.46 | Y |
| Gastrointestinal disorders | Immune-mediated pancreatitis | 30 | 0.000517774 | Y | 1.11 | 0.59 | 1.62 | Y |
| Gastrointestinal disorders | Duodenitis | 18 | 0.000233799 | Y | 0.73 | 0.07 | 1.4 | Y |
| Gastrointestinal disorders | Oesophageal achalasia | 3 | 0.000104016 | Y | 2.16 | 0.53 | 3.79 | Y |
| Gastrointestinal disorders | Oesophageal haemorrhage | 3 | 0.000114352 | Y | 1.66 | 0.03 | 3.29 | Y |
| Gastrointestinal disorders | Eosinophilic gastritis | 3 | 9.54773E-05 | Y | 2.77 | 1.14 | 4.4 | Y |
| Gastrointestinal disorders | Salivary hyposecretion | 4 | 0.000141288 | Y | 2.23 | 0.82 | 3.64 | Y |
| Gastrointestinal disorders | Gastrointestinal toxicity | 43 | 0.000708765 | Y | 1.04 | 0.61 | 1.47 | Y |
| Gastrointestinal disorders | Salivary duct inflammation | 2 | 6.36623E-05 | Y | 2.3 | 0.3 | 4.3 | Y |
| Gastrointestinal disorders | Haemorrhoids thrombosed | 3 | 9.92009E-05 | Y | 2.47 | 0.84 | 4.1 | Y |
| General disorders and administration site conditions | Necrosis | 9 | 0.000216523 | Y | 1.65 | 0.71 | 2.6 | Y |
| General disorders and administration site conditions | Thirst | 17 | 0.000368931 | Y | 0.73 | 0.04 | 1.41 | Y |
| General disorders and administration site conditions | Oedema mucosal | 3 | 0.000108349 | Y | 1.93 | 0.3 | 3.56 | Y |
| Hepatobiliary disorders | Hepatic function abnormal | 174 | 0.002436692 | Y | 0.84 | 0.63 | 1.05 | Y |
| Hepatobiliary disorders | Hepatic cytolysis | 38 | 0.001050498 | Y | 2.85 | 2.39 | 3.31 | Y |
| Hepatobiliary disorders | Liver disorder | 200 | 0.002285094 | Y | 0.64 | 0.45 | 0.84 | Y |
| Hepatobiliary disorders | Hypertransaminasaemia | 16 | 0.000262931 | Y | 1.01 | 0.3 | 1.72 | Y |
| Hepatobiliary disorders | Immune-mediated hepatitis | 151 | 0.003225191 | Y | 1.61 | 1.38 | 1.84 | Y |
| Hepatobiliary disorders | Immune-mediated hepatic disorder | 143 | 0.00311877 | Y | 1.67 | 1.43 | 1.91 | Y |
| Hepatobiliary disorders | Granulomatous liver disease | 7 | 0.000215043 | Y | 2.9 | 1.83 | 3.97 | Y |
| Hepatobiliary disorders | Subacute hepatic failure | 2 | 6.50104E-05 | Y | 2.18 | 0.18 | 4.18 | Y |
| Hepatobiliary disorders | Drug-induced liver injury | 46 | 0.000496429 | Y | 0.59 | 0.18 | 1.01 | Y |
| Immune system disorders | Haemophagocytic lymphohistiocytosis | 54 | 0.000977471 | Y | 1.2 | 0.82 | 1.59 | Y |
| Immune system disorders | Cytokine storm | 5 | 0.000150867 | Y | 2.45 | 1.19 | 3.72 | Y |
| Immune system disorders | Cytokine release syndrome | 122 | 0.003119908 | Y | 2.35 | 2.09 | 2.61 | Y |
| Infections and infestations | Meningitis aseptic | 52 | 0.000558358 | Y | 0.59 | 0.2 | 0.98 | Y |
| Infections and infestations | Infectious pleural effusion | 11 | 0.000188281 | Y | 0.93 | 0.08 | 1.78 | Y |
| Infections and infestations | Coronavirus infection | 9 | 0.000293756 | Y | 0.99 | 0.04 | 1.93 | Y |
| Infections and infestations | Large intestine infection | 4 | 0.000131474 | Y | 1.97 | 0.55 | 3.38 | Y |
| Infections and infestations | Pyelonephritis acute | 4 | 0.000143888 | Y | 2.11 | 0.7 | 3.53 | Y |
| Infections and infestations | Cytomegalovirus infection reactivation | 4 | 0.000143631 | Y | 2.12 | 0.71 | 3.54 | Y |
| Infections and infestations | Hepatitis viral | 3 | 8.85847E-05 | Y | 1.8 | 0.17 | 3.44 | Y |
| Infections and infestations | Epiglottitis | 3 | 0.000102892 | Y | 2.23 | 0.6 | 3.86 | Y |
| Infections and infestations | Pseudomonal sepsis | 3 | 0.000106102 | Y | 2.04 | 0.41 | 3.68 | Y |
| Infections and infestations | Cytomegalovirus oesophagitis | 3 | 8.22611E-05 | Y | 1.8 | 0.17 | 3.44 | Y |
| Infections and infestations | Encephalitis brain stem | 3 | 9.67292E-05 | Y | 2.66 | 1.03 | 4.3 | Y |
| Infections and infestations | Parotitis | 3 | 9.18267E-05 | Y | 1.8 | 0.17 | 3.44 | Y |
| Infections and infestations | Adenovirus infection | 3 | 9.29823E-05 | Y | 1.8 | 0.17 | 3.44 | Y |
| Infections and infestations | Cytomegalovirus hepatitis | 2 | 6.61981E-05 | Y | 2.09 | 0.09 | 4.08 | Y |
| Infections and infestations | Mucosal infection | 2 | 6.55561E-05 | Y | 2.14 | 0.14 | 4.14 | Y |
| Injury, poisoning and procedural complications | Radiation cataract | 2 | 6.33734E-05 | Y | 2.32 | 0.32 | 4.32 | Y |
| Injury, poisoning and procedural complications | Acetabulum fracture | 2 | 6.72253E-05 | Y | 2.01 | 0.01 | 4.01 | Y |
| Investigations | Alanine aminotransferase | 2 | 6.60697E-05 | Y | 2.1 | 0.1 | 4.09 | Y |
| Investigations | Troponin I increased | 9 | 0.000189842 | Y | 1.24 | 0.3 | 2.18 | Y |
| Investigations | Thyroid hormones decreased | 4 | 0.000121844 | Y | 2.17 | 0.75 | 3.58 | Y |
| Investigations | Granulocyte count increased | 2 | 6.65833E-05 | Y | 2.06 | 0.06 | 4.06 | Y |
| Investigations | Lymphocyte count increased | 5 | 0.000172125 | Y | 1.5 | 0.24 | 2.77 | Y |
| Investigations | Enzyme level increased | 3 | 9.45552E-05 | Y | 1.8 | 0.17 | 3.44 | Y |
| Investigations | Aspartate aminotransferase | 3 | 9.73391E-05 | Y | 2.61 | 0.98 | 4.25 | Y |
| Investigations | Blood corticotrophin increased | 7 | 0.000145037 | Y | 1.31 | 0.25 | 2.38 | Y |
| Investigations | Blood lactate dehydrogenase abnormal | 4 | 0.000121363 | Y | 2.17 | 0.75 | 3.58 | Y |
| Metabolism and nutrition disorders | Type 1 diabetes mellitus | 148 | 0.001827678 | Y | 0.71 | 0.48 | 0.94 | Y |
| Metabolism and nutrition disorders | Latent autoimmune diabetes in adults | 2 | 6.57808E-05 | Y | 2.12 | 0.12 | 4.12 | Y |
| Metabolism and nutrition disorders | Hyperglycaemia | 55 | 0.000414233 | Y | 0.39 | 0.01 | 0.77 | Y |
| Metabolism and nutrition disorders | Hyperlipasaemia | 4 | 9.92872E-05 | Y | 1.58 | 0.16 | 2.99 | Y |
| Metabolism and nutrition disorders | Steroid diabetes | 4 | 0.00013689 | Y | 2.46 | 1.04 | 3.87 | Y |
| Metabolism and nutrition disorders | Acidosis | 11 | 0.000271784 | Y | 1.71 | 0.86 | 2.56 | Y |
| Metabolism and nutrition disorders | Diabetic ketoacidosis | 154 | 0.002173387 | Y | 0.85 | 0.62 | 1.07 | Y |
| Metabolism and nutrition disorders | Weight fluctuation | 12 | 0.000304931 | Y | 1.33 | 0.51 | 2.15 | Y |
| Musculoskeletal and connective tissue disorders | Arthritis | 111 | 0.000652041 | Y | 0.29 | 0.03 | 0.56 | Y |
| Musculoskeletal and connective tissue disorders | Rhabdomyolysis | 48 | 0.000830298 | Y | 0.66 | 0.25 | 1.07 | Y |
| Musculoskeletal and connective tissue disorders | Musculoskeletal toxicity | 3 | 9.7307E-05 | Y | 2.62 | 0.98 | 4.25 | Y |
| Musculoskeletal and connective tissue disorders | Myositis | 120 | 0.001277643 | Y | 0.59 | 0.33 | 0.85 | Y |
| Musculoskeletal and connective tissue disorders | Immune-mediated myositis | 51 | 0.000957662 | Y | 1.19 | 0.79 | 1.58 | Y |
| Musculoskeletal and connective tissue disorders | Sjogren's syndrome | 41 | 0.000419035 | Y | 0.55 | 0.11 | 1 | Y |
| Neoplasms benign, malignant and unspecified | Non-small cell lung cancer | 92 | 0.002759166 | Y | 3.62 | 3.32 | 3.91 | Y |
| Neoplasms benign, malignant and unspecified | Neoplasm recurrence | 12 | 0.000350624 | Y | 2.05 | 1.23 | 2.87 | Y |
| Neoplasms benign, malignant and unspecified | Tumour invasion | 4 | 0.000132813 | Y | 2.7 | 1.29 | 4.12 | Y |
| Neoplasms benign, malignant and unspecified | Non-small cell lung cancer metastatic | 3 | 0.000107354 | Y | 1.98 | 0.35 | 3.61 | Y |
| Neoplasms benign, malignant and unspecified | Pleural mesothelioma malignant | 2 | 6.52351E-05 | Y | 2.16 | 0.16 | 4.16 | Y |
| Nervous system disorders | Polyneuropathy | 25 | 0.000277573 | Y | 0.61 | 0.04 | 1.17 | Y |
| Nervous system disorders | Myelitis transverse | 12 | 0.00023648 | Y | 1.31 | 0.5 | 2.13 | Y |
| Nervous system disorders | Acute motor-sensory axonal neuropathy | 2 | 6.63265E-05 | Y | 2.08 | 0.08 | 4.07 | Y |
| Nervous system disorders | Acute motor axonal neuropathy | 3 | 8.03352E-05 | Y | 1.8 | 0.17 | 3.44 | Y |
| Nervous system disorders | Immune-mediated neuropathy | 7 | 0.000159441 | Y | 1.58 | 0.51 | 2.65 | Y |
| Nervous system disorders | Immune-mediated encephalitis | 30 | 0.000480963 | Y | 0.99 | 0.48 | 1.51 | Y |
| Nervous system disorders | Immune-mediated neurological disorder | 6 | 0.000158873 | Y | 2.11 | 0.95 | 3.26 | Y |
| Nervous system disorders | Immune-mediated optic neuritis | 4 | 9.49216E-05 | Y | 1.58 | 0.16 | 2.99 | Y |
| Nervous system disorders | Immune-mediated myasthenia gravis | 26 | 0.000571051 | Y | 1.63 | 1.08 | 2.19 | Y |
| Nervous system disorders | Hydrocephalus | 6 | 0.000165381 | Y | 1.17 | 0.01 | 2.32 | Y |
| Nervous system disorders | Cerebral mass effect | 3 | 9.096E-05 | Y | 1.8 | 0.17 | 3.44 | Y |
| Nervous system disorders | Bulbar palsy | 2 | 6.66475E-05 | Y | 2.05 | 0.05 | 4.05 | Y |
| Nervous system disorders | Depressed level of consciousness | 29 | 0.000693332 | Y | 0.92 | 0.39 | 1.44 | Y |
| Nervous system disorders | Axonal and demyelinating polyneuropathy | 2 | 6.4561E-05 | Y | 2.22 | 0.22 | 4.22 | Y |
| Nervous system disorders | Autonomic neuropathy | 4 | 0.000105996 | Y | 1.58 | 0.16 | 2.99 | Y |
| Renal and urinary disorders | Glomerulonephritis rapidly progressive | 9 | 0.000236071 | Y | 1.92 | 0.98 | 2.86 | Y |
| Renal and urinary disorders | Immune-mediated nephritis | 28 | 0.000501913 | Y | 1.17 | 0.64 | 1.71 | Y |
| Renal and urinary disorders | Immune-mediated renal disorder | 14 | 0.000239383 | Y | 1.04 | 0.29 | 1.8 | Y |
| Renal and urinary disorders | Urinary tract obstruction | 8 | 0.000210235 | Y | 1.49 | 0.5 | 2.49 | Y |
| Renal and urinary disorders | Kidney congestion | 2 | 6.50746E-05 | Y | 2.17 | 0.18 | 4.17 | Y |
| Renal and urinary disorders | Nephropathy toxic | 20 | 0.000389166 | Y | 1.03 | 0.39 | 1.66 | Y |
| Reproductive system and breast disorders | Spermatogenesis abnormal | 4 | 0.000128512 | Y | 3.02 | 1.6 | 4.43 | Y |
| Respiratory, thoracic and mediastinal disorders | Paranasal sinus inflammation | 2 | 6.56845E-05 | Y | 2.13 | 0.13 | 4.12 | Y |
| Respiratory, thoracic and mediastinal disorders | Non-cardiogenic pulmonary oedema | 3 | 9.43626E-05 | Y | 1.8 | 0.17 | 3.44 | Y |
| Respiratory, thoracic and mediastinal disorders | Pulmonary artery thrombosis | 4 | 0.000103203 | Y | 1.58 | 0.16 | 2.99 | Y |
| Respiratory, thoracic and mediastinal disorders | Pulmonary toxicity | 57 | 0.000900532 | Y | 0.98 | 0.61 | 1.36 | Y |
| Respiratory, thoracic and mediastinal disorders | Diffuse panbronchiolitis | 2 | 6.40154E-05 | Y | 2.26 | 0.27 | 4.26 | Y |
| Respiratory, thoracic and mediastinal disorders | Chylothorax | 6 | 0.000151531 | Y | 1.69 | 0.54 | 2.85 | Y |
| Respiratory, thoracic and mediastinal disorders | Pleural disorder | 3 | 0.00010225 | Y | 2.27 | 0.63 | 3.9 | Y |
| Respiratory, thoracic and mediastinal disorders | Autoimmune lung disease | 6 | 0.000159162 | Y | 2.11 | 0.95 | 3.26 | Y |
| Respiratory, thoracic and mediastinal disorders | Mediastinal haemorrhage | 2 | 6.49141E-05 | Y | 2.19 | 0.19 | 4.19 | Y |
| Skin and subcutaneous tissue disorders | Erythema multiforme | 31 | 0.000755376 | Y | 1.44 | 0.94 | 1.95 | Y |
| Skin and subcutaneous tissue disorders | Achromotrichia acquired | 7 | 0.00020644 | Y | 2.9 | 1.83 | 3.97 | Y |
| Skin and subcutaneous tissue disorders | Hair colour changes | 11 | 0.00027795 | Y | 1.06 | 0.2 | 1.91 | Y |
| Skin and subcutaneous tissue disorders | Immune-mediated dermatitis | 90 | 0.002352457 | Y | 2.48 | 2.18 | 2.78 | Y |
| Skin and subcutaneous tissue disorders | Skin disorder | 66 | 0.000528493 | Y | 0.42 | 0.07 | 0.76 | Y |
| Skin and subcutaneous tissue disorders | Stevens-Johnson syndrome | 48 | 0.000645754 | Y | 0.79 | 0.38 | 1.19 | Y |
| Skin and subcutaneous tissue disorders | Drug eruption | 24 | 0.000481853 | Y | 0.85 | 0.27 | 1.43 | Y |
| Skin and subcutaneous tissue disorders | Toxic epidermal necrolysis | 40 | 0.000677176 | Y | 1.08 | 0.64 | 1.53 | Y |
| Surgical and medical procedures | Probiotic therapy | 2 | 6.47536E-05 | Y | 2.2 | 0.2 | 4.2 | Y |
| Vascular disorders | Immune-mediated vasculitis | 3 | 9.51884E-05 | Y | 2.8 | 1.16 | 4.43 | Y |
| Vascular disorders | Shock | 19 | 0.000329158 | Y | 0.78 | 0.13 | 1.43 | Y |

Table 7 Ω shrinkage measure models, ROR and IC methods for signal detection

| SOC | PT | Frequency | ROR | ROR_025_ | ROR_975_ | IC | IC_025_ | IC_975_ | Ω | Ω025 | Ω095 |
| --- | --- | --- | --- | --- | --- | --- | --- | --- | --- | --- | --- |
| Endocrine disorders | Pituitary infarction | 3 | 97.63 | 30.14 | 316.28 | 6.5 | 0.44 | 3.46 | 2.78 | 1.15 | 4.41 |
| Infections and infestations | Large intestine infection | 7 | 6.28 | 2.99 | 13.19 | 2.64 | 0.89 | 2.94 | 1.97 | 0.55 | 3.38 |
| Endocrine disorders | Polyglandular disorder | 3 | 71.34 | 22.28 | 228.46 | 6.08 | 0.44 | 3.43 | 2.74 | 1.11 | 4.37 |
| Nervous system disorders | Myelitis transverse | 12 | 12.29 | 6.96 | 21.7 | 3.61 | 1.91 | 3.51 | 1.31 | 0.5 | 2.13 |
| Musculoskeletal and connective tissue disorders | Musculoskeletal toxicity | 3 | 26.88 | 8.56 | 84.38 | 4.72 | 0.38 | 3.31 | 2.62 | 0.98 | 4.25 |
| Nervous system disorders | Acute motor axonal neuropathy | 3 | 48.81 | 15.4 | 154.75 | 5.55 | 0.43 | 3.39 | 1.8 | 0.17 | 3.44 |
| Eye disorders | Corneal perforation | 3 | 7 | 2.25 | 21.77 | 2.8 | 0.03 | 2.93 | 1.8 | 0.17 | 3.44 |
| Reproductive system and breast disorders | Spermatogenesis abnormal | 4 | 64.24 | 23.51 | 175.53 | 5.93 | 0.9 | 3.56 | 3.02 | 1.6 | 4.43 |
| Eye disorders | Choroidal effusion | 4 | 7.69 | 2.88 | 20.56 | 2.94 | 0.42 | 3.01 | 1.8 | 0.17 | 3.44 |
| Hepatobiliary disorders | Immune-mediated hepatitis | 181 | 205.8 | 175.8 | 240.93 | 7.46 | 6.26 | 6.72 | 1.61 | 1.38 | 1.84 |
| Endocrine disorders | Immune-mediated thyroiditis | 50 | 275.1 | 202.46 | 373.8 | 7.81 | 4.94 | 5.82 | 1.45 | 1.07 | 1.83 |
| Nervous system disorders | Immune-mediated neurological disorder | 5 | 137.41 | 54.54 | 346.18 | 6.95 | 1.28 | 3.77 | 2.11 | 0.95 | 3.26 |
| Blood and lymphatic system disorders | Immune-mediated cytopenia | 6 | 164.9 | 70.35 | 386.53 | 7.19 | 1.59 | 3.91 | 2.17 | 0.75 | 3.58 |
| Endocrine disorders | Endocrine toxicity | 23 | 557.95 | 341 | 912.92 | 8.59 | 3.82 | 5.18 | 4.55 | 3.96 | 5.14 |
| Renal and urinary disorders | Urinary tract obstruction | 11 | 5.43 | 3 | 9.81 | 2.43 | 1.15 | 2.82 | 1.49 | 0.5 | 2.49 |
| Hepatobiliary disorders | Granulomatous liver disease | 6 | 11.58 | 5.18 | 25.86 | 3.52 | 1.1 | 3.3 | 2.9 | 1.83 | 3.97 |
| Endocrine disorders | Glucocorticoid deficiency | 17 | 69.86 | 42.86 | 113.89 | 6.05 | 3.14 | 4.54 | 1.65 | 0.7 | 2.59 |
| Investigations | Aspartate aminotransferase | 3 | 17.26 | 5.52 | 53.93 | 4.09 | 0.31 | 3.22 | 2.61 | 0.98 | 4.25 |
| Gastrointestinal disorders | Gastrointestinal toxicity | 48 | 15.87 | 11.94 | 21.11 | 3.97 | 3.18 | 4.01 | 1.04 | 0.61 | 1.47 |
| Investigations | Blood lactate dehydrogenase abnormal | 4 | 8.74 | 3.27 | 23.37 | 3.12 | 0.48 | 3.07 | 2.17 | 0.75 | 3.58 |
| Gastrointestinal disorders | Haemorrhoids thrombosed | 3 | 14 | 4.49 | 43.69 | 3.79 | 0.26 | 3.17 | 2.47 | 0.84 | 4.1 |
| Neoplasms benign, malignant and unspecified | Neoplasm recurrence | 12 | 4.96 | 2.81 | 8.74 | 2.3 | 1.12 | 2.72 | 2.05 | 1.23 | 2.87 |
| Eye disorders | Autoimmune uveitis | 5 | 114.51 | 45.8 | 286.26 | 6.71 | 1.28 | 3.75 | 2.17 | 0.75 | 3.58 |
| Eye disorders | Autoimmune retinopathy | 3 | 92.75 | 28.69 | 299.82 | 6.43 | 0.44 | 3.46 | 1.8 | 0.17 | 3.44 |
| Respiratory, thoracic and mediastinal disorders | Autoimmune lung disease | 7 | 139.63 | 63.91 | 305.08 | 6.97 | 1.84 | 4 | 2.11 | 0.95 | 3.26 |

Table 8 Shrinkage measure model signals

| SOC | PT | Frequency | Ω | Ω025 | Ω095 |
| --- | --- | --- | --- | --- | --- |
| Endocrine disorders | Hyperthyroidism | 162 | 0.5 | 0.28 | 0.73 |
|  | Thyroiditis | 122 | 0.48 | 0.22 | 0.73 |
|  | Secondary adrenocortical insufficiency | 74 | 1.18 | 0.85 | 1.51 |
|  | Immune-mediated hypophysitis | 63 | 1.51 | 1.15 | 1.86 |
|  | Adrenocorticotropic hormone deficiency | 62 | 1.25 | 0.89 | 1.61 |
|  | Immune-mediated thyroiditis | 56 | 1.45 | 1.07 | 1.83 |
|  | Immune-mediated hypothyroidism | 46 | 0.79 | 0.37 | 1.21 |
|  | Immune-mediated adrenal insufficiency | 45 | 1.79 | 1.37 | 2.21 |
|  | Endocrine toxicity | 23 | 4.55 | 3.96 | 5.14 |
|  | Thyrotoxic crisis | 15 | 0.95 | 0.22 | 1.68 |
|  | Immune-mediated endocrinopathy | 14 | 3.27 | 2.51 | 4.02 |
|  | Immune-mediated hyperthyroidism | 14 | 2.53 | 1.77 | 3.28 |
|  | Glucocorticoid deficiency | 9 | 1.65 | 0.7 | 2.59 |
|  | Hypoparathyroidism | 6 | 1.37 | 0.22 | 2.53 |
|  | Primary adrenal insufficiency | 6 | 1.37 | 0.22 | 2.53 |
|  | Pituitary infarction | 3 | 2.78 | 1.15 | 4.41 |
|  | Thyroid stimulating hormone deficiency | 3 | 2.78 | 1.14 | 4.41 |
|  | Polyglandular disorder | 3 | 2.74 | 1.11 | 4.37 |
|  | Thyroiditis acute | 3 | 2.61 | 0.98 | 4.24 |
|  | Primary hypoparathyroidism | 2 | 2.3 | 0.3 | 4.3 |
| Nervous system disorders | Immune-mediated encephalitis | 30 | 0.99 | 0.48 | 1.51 |
|  | Depressed level of consciousness | 29 | 0.92 | 0.39 | 1.44 |
|  | Immune-mediated myasthenia gravis | 26 | 1.63 | 1.08 | 2.19 |
|  | Polyneuropathy | 25 | 0.61 | 0.04 | 1.17 |
|  | Myelitis transverse | 12 | 1.31 | 0.5 | 2.13 |
|  | Immune-mediated neuropathy | 7 | 1.58 | 0.51 | 2.65 |
|  | Immune-mediated neurological disorder | 6 | 2.11 | 0.95 | 3.26 |
|  | Hydrocephalus | 6 | 1.17 | 0.01 | 2.32 |
|  | Immune-mediated optic neuritis | 4 | 1.58 | 0.16 | 2.99 |
|  | Autonomic neuropathy | 4 | 1.58 | 0.16 | 2.99 |
|  | Acute motor axonal neuropathy | 3 | 1.8 | 0.17 | 3.44 |
|  | Cerebral mass effect | 3 | 1.8 | 0.17 | 3.44 |
|  | Acute motor-sensory axonal neuropathy | 2 | 2.08 | 0.08 | 4.07 |
|  | Bulbar palsy | 2 | 2.05 | 0.05 | 4.05 |
|  | Axonal and demyelinating polyneuropathy | 2 | 2.22 | 0.22 | 4.22 |
| Infections and infestations | Meningitis aseptic | 52 | 0.59 | 0.2 | 0.98 |
|  | Infectious pleural effusion | 11 | 0.93 | 0.08 | 1.78 |
|  | Coronavirus infection | 9 | 0.99 | 0.04 | 1.93 |
|  | Large intestine infection | 4 | 1.97 | 0.55 | 3.38 |
|  | Pyelonephritis acute | 4 | 2.11 | 0.7 | 3.53 |
|  | Cytomegalovirus infection reactivation | 4 | 2.12 | 0.71 | 3.54 |
|  | Hepatitis viral | 3 | 1.8 | 0.17 | 3.44 |
|  | Epiglottitis | 3 | 2.23 | 0.6 | 3.86 |
|  | Pseudomonal sepsis | 3 | 2.04 | 0.41 | 3.68 |
|  | Cytomegalovirus oesophagitis | 3 | 1.8 | 0.17 | 3.44 |
|  | Encephalitis brain stem | 3 | 2.66 | 1.03 | 4.3 |
|  | Parotitis | 3 | 1.8 | 0.17 | 3.44 |
|  | Adenovirus infection | 3 | 1.8 | 0.17 | 3.44 |
|  | Cytomegalovirus hepatitis | 2 | 2.09 | 0.09 | 4.08 |
|  | Mucosal infection | 2 | 2.14 | 0.14 | 4.14 |
| Gastrointestinal disorders | Immune-mediated enterocolitis | 395 | 0.32 | 0.17 | 0.46 |
|  | Gastrointestinal toxicity | 43 | 1.04 | 0.61 | 1.47 |
|  | Immune-mediated pancreatitis | 30 | 1.11 | 0.59 | 1.62 |
|  | Duodenitis | 18 | 0.73 | 0.07 | 1.4 |
|  | Gastritis haemorrhagic | 7 | 1.31 | 0.25 | 2.38 |
|  | Salivary hyposecretion | 4 | 2.23 | 0.82 | 3.64 |
|  | Malignant ascites | 3 | 1.8 | 0.17 | 3.44 |
|  | Oesophageal achalasia | 3 | 2.16 | 0.53 | 3.79 |
|  | Oesophageal haemorrhage | 3 | 1.66 | 0.03 | 3.29 |
|  | Eosinophilic gastritis | 3 | 2.77 | 1.14 | 4.4 |
|  | Haemorrhoids thrombosed | 3 | 2.47 | 0.84 | 4.1 |
|  | Proctitis haemorrhagic | 2 | 2.23 | 0.23 | 4.23 |
|  | Salivary duct inflammation | 2 | 2.3 | 0.3 | 4.3 |
| Cardiac disorders | Myocarditis | 195 | 0.54 | 0.33 | 0.74 |
|  | Immune-mediated myocarditis | 125 | 1.03 | 0.77 | 1.28 |
|  | Acute myocardial infarction | 24 | 0.65 | 0.07 | 1.23 |
|  | Cardiogenic shock | 23 | 1.22 | 0.63 | 1.81 |
|  | Sinus tachycardia | 18 | 0.95 | 0.28 | 1.61 |
|  | Ventricular tachycardia | 13 | 0.94 | 0.15 | 1.72 |
|  | Ventricular arrhythmia | 5 | 1.45 | 0.19 | 2.72 |
|  | Bundle branch block right | 5 | 1.42 | 0.16 | 2.69 |
|  | Carditis | 3 | 2.35 | 0.72 | 3.99 |
|  | Cardiac sarcoidosis | 2 | 2.1 | 0.1 | 4.1 |
| Respiratory disorders | Pulmonary toxicity | 57 | 0.98 | 0.61 | 1.36 |
|  | Chylothorax | 6 | 1.69 | 0.54 | 2.85 |
|  | Autoimmune lung disease | 6 | 2.11 | 0.95 | 3.26 |
|  | Pulmonary artery thrombosis | 4 | 1.58 | 0.16 | 2.99 |
|  | Non-cardiogenic pulmonary oedema | 3 | 1.8 | 0.17 | 3.44 |
|  | Pleural disorder | 3 | 2.27 | 0.63 | 3.9 |
|  | Paranasal sinus inflammation | 2 | 2.13 | 0.13 | 4.12 |
|  | Diffuse panbronchiolitis | 2 | 2.26 | 0.27 | 4.26 |
|  | Mediastinal haemorrhage | 2 | 2.19 | 0.19 | 4.19 |
| Hepatobiliary disorders | Liver disorder | 200 | 0.64 | 0.45 | 0.84 |
|  | Hepatic function abnormal | 174 | 0.84 | 0.63 | 1.05 |
|  | Immune-mediated hepatitis | 151 | 1.61 | 1.38 | 1.84 |
|  | Immune-mediated hepatic disorder | 143 | 1.67 | 1.43 | 1.91 |
|  | Drug-induced liver injury | 46 | 0.59 | 0.18 | 1.01 |
|  | Hepatic cytolysis | 38 | 2.85 | 2.39 | 3.31 |
|  | Hypertransaminasaemia | 16 | 1.01 | 0.3 | 1.72 |
|  | Granulomatous liver disease | 7 | 2.9 | 1.83 | 3.97 |
|  | Subacute hepatic failure | 2 | 2.18 | 0.18 | 4.18 |
| Skin and subcutaneous tissue disorders | Immune-mediated dermatitis | 90 | 2.48 | 2.18 | 2.78 |
|  | Skin disorder | 66 | 0.42 | 0.07 | 0.76 |
|  | Stevens-Johnson syndrome | 48 | 0.79 | 0.38 | 1.19 |
|  | Toxic epidermal necrolysis | 40 | 1.08 | 0.64 | 1.53 |
|  | Erythema multiforme | 31 | 1.44 | 0.94 | 1.95 |
|  | Drug eruption | 24 | 0.85 | 0.27 | 1.43 |
|  | Hair colour changes | 11 | 1.06 | 0.2 | 1.91 |
|  | Achromotrichia acquired | 7 | 2.9 | 1.83 | 3.97 |
| Metabolism and nutrition disorders | Diabetic ketoacidosis | 154 | 0.85 | 0.62 | 1.07 |
|  | Type 1 diabetes mellitus | 148 | 0.71 | 0.48 | 0.94 |
|  | Hyperglycaemia | 55 | 0.39 | 0.01 | 0.77 |
|  | Weight fluctuation | 12 | 1.33 | 0.51 | 2.15 |
|  | Acidosis | 11 | 1.71 | 0.86 | 2.56 |
|  | Hyperlipasaemia | 4 | 1.58 | 0.16 | 2.99 |
|  | Steroid diabetes | 4 | 2.46 | 1.04 | 3.87 |
|  | Latent autoimmune diabetes in adults | 2 | 2.12 | 0.12 | 4.12 |
| Blood and lymphatic system disorders | Febrile neutropenia | 32 | 0.77 | 0.27 | 1.27 |
|  | Splenomegaly | 10 | 1.01 | 0.12 | 1.91 |
|  | Pseudolymphoma | 4 | 2.6 | 1.18 | 4.01 |
|  | Immune-mediated cytopenia | 4 | 2.17 | 0.75 | 3.58 |
|  | Immune-mediated pancytopenia | 3 | 2.78 | 1.15 | 4.41 |
|  | Bone marrow infiltration | 2 | 2.02 | 0.02 | 4.02 |
|  | Hyposplenism | 2 | 2.3 | 0.3 | 4.3 |
| Eye disorders | Immune-mediated uveitis | 34 | 0.75 | 0.26 | 1.23 |
|  | Hypotony of eye | 4 | 2.64 | 1.22 | 4.05 |
|  | Autoimmune uveitis | 4 | 2.17 | 0.75 | 3.58 |
|  | Corneal perforation | 3 | 1.8 | 0.17 | 3.44 |
|  | Choroidal effusion | 3 | 1.8 | 0.17 | 3.44 |
|  | Autoimmune retinopathy | 3 | 1.8 | 0.17 | 3.44 |
| Renal and urinary disorders | Immune-mediated nephritis | 28 | 1.17 | 0.64 | 1.71 |
|  | Nephropathy toxic | 20 | 1.03 | 0.39 | 1.66 |
|  | Immune-mediated renal disorder | 14 | 1.04 | 0.29 | 1.8 |
|  | Glomerulonephritis rapidly progressive | 9 | 1.92 | 0.98 | 2.86 |
|  | Urinary tract obstruction | 8 | 1.49 | 0.5 | 2.49 |
|  | Kidney congestion | 2 | 2.17 | 0.18 | 4.17 |
| Musculoskeletal and connective tissue disorders | Myositis | 120 | 0.59 | 0.33 | 0.85 |
|  | Arthritis | 111 | 0.29 | 0.03 | 0.56 |
|  | Immune-mediated myositis | 51 | 1.19 | 0.79 | 1.58 |
|  | Rhabdomyolysis | 48 | 0.66 | 0.25 | 1.07 |
|  | Sjogren's syndrome | 41 | 0.55 | 0.11 | 1 |
|  | Musculoskeletal toxicity | 3 | 2.62 | 0.98 | 4.25 |
| Immune system disorders | Cytokine release syndrome | 122 | 2.35 | 2.09 | 2.61 |
|  | Haemophagocytic lymphohistiocytosis | 54 | 1.2 | 0.82 | 1.59 |
|  | Cytokine storm | 5 | 2.45 | 1.19 | 3.72 |
| Vascular disorders | Shock | 19 | 0.78 | 0.13 | 1.43 |
|  | Immune-mediated vasculitis | 3 | 2.8 | 1.16 | 4.43 |
| Reproductive system and breast disorders | Spermatogenesis abnormal | 4 | 3.02 | 1.6 | 4.43 |
| Ear and labyrinth disorders | Vestibular disorder | 4 | 1.58 | 0.16 | 2.99 |

Table 9 Sensitivity analysis stratified by reporter type (Healthcare Professionals)

| SOC | PT | Frequency | ROR | ROR_025_ | ROR_975_ | ROR_Signal | IC | ICIC_025_ | IC_975_ | IC_Signal |
| --- | --- | --- | --- | --- | --- | --- | --- | --- | --- | --- |
| Eye disorders | Iridocyclitis | 28 | 9.79 | 6.74 | 14.21 | Y | 3.27 | 2.36 | 3.43 | Y |
| Eye disorders | Iris adhesions | 4 | 8.36 | 3.12 | 22.4 | Y | 3.05 | 0.45 | 3.05 | Y |
| Eye disorders | Corneal perforation | 2 | 3.74 | 0.93 | 15.02 | N | 1.9 | -0.71 | 2.64 | N |
| Eye disorders | Choroidal effusion | 2 | 2.83 | 0.7 | 11.33 | N | 1.5 | -0.86 | 2.48 | N |
| Eye disorders | Orbital myositis | 5 | 35.93 | 14.65 | 88.13 | Y | 5.1 | 1.18 | 3.6 | Y |
| Eye disorders | Autoimmune uveitis | 5 | 89.82 | 35.54 | 227.04 | Y | 6.33 | 1.25 | 3.75 | Y |
| Eye disorders | Autoimmune retinopathy | 3 | 83.83 | 25.43 | 276.34 | Y | 6.24 | 0.41 | 3.48 | Y |
| Nervous system disorders | Myelitis transverse | 11 | 9.99 | 5.51 | 18.11 | Y | 3.3 | 1.66 | 3.34 | Y |
| Nervous system disorders | Acute motor axonal neuropathy | 3 | 32.8 | 10.32 | 104.22 | Y | 4.98 | 0.39 | 3.35 | Y |
| Nervous system disorders | Miller Fisher syndrome | 4 | 20.25 | 7.5 | 54.68 | Y | 4.3 | 0.74 | 3.37 | Y |
| Nervous system disorders | Immune-mediated neurological disorder | 5 | 87.73 | 34.75 | 221.51 | Y | 6.3 | 1.25 | 3.74 | Y |
| Nervous system disorders | Meningoradiculitis | 9 | 93.03 | 46.55 | 185.95 | Y | 6.37 | 2.21 | 4.14 | Y |
| Nervous system disorders | Radiculopathy | 14 | 5.7 | 3.37 | 9.65 | Y | 2.5 | 1.36 | 2.86 | Y |
| Nervous system disorders | Peripheral sensorimotor neuropathy | 8 | 7.64 | 3.81 | 15.33 | Y | 2.92 | 1.16 | 3.1 | Y |
| Gastrointestinal disorders | Colonic fistula | 4 | 11.88 | 4.42 | 31.9 | Y | 3.55 | 0.59 | 3.2 | Y |
| Gastrointestinal disorders | Oral lichenoid reaction | 3 | 48.16 | 14.99 | 154.73 | Y | 5.5 | 0.41 | 3.4 | Y |
| Gastrointestinal disorders | Colitis erosive | 3 | 21.35 | 6.78 | 67.27 | Y | 4.38 | 0.34 | 3.27 | Y |
| Gastrointestinal disorders | Gastrointestinal toxicity | 45 | 10.25 | 7.63 | 13.75 | Y | 3.34 | 2.65 | 3.51 | Y |
| Gastrointestinal disorders | Haemorrhoids thrombosed | 3 | 15.72 | 5.01 | 49.31 | Y | 3.95 | 0.28 | 3.2 | Y |
| Gastrointestinal disorders | Pancreatic toxicity | 6 | 64.67 | 28.09 | 148.89 | Y | 5.9 | 1.53 | 3.81 | Y |
| Endocrine disorders | Pituitary infarction | 3 | 73.01 | 22.32 | 238.84 | Y | 6.06 | 0.41 | 3.46 | Y |
| Endocrine disorders | Polyglandular disorder | 3 | 80.84 | 24.57 | 265.91 | Y | 6.19 | 0.41 | 3.47 | Y |
| Endocrine disorders | Immune-mediated thyroiditis | 49 | 167.5 | 122.89 | 228.3 | Y | 7.1 | 4.76 | 5.65 | Y |
| Endocrine disorders | Endocrine toxicity | 23 | 434.07 | 259.87 | 725.03 | Y | 8.11 | 3.78 | 5.16 | Y |
| Endocrine disorders | Glucocorticoid deficiency | 16 | 51.39 | 30.97 | 85.28 | Y | 5.59 | 2.95 | 4.4 | Y |
| Blood and lymphatic system disorders | Cold type haemolytic anaemia | 3 | 14.89 | 4.75 | 46.69 | Y | 3.87 | 0.27 | 3.19 | Y |
| Blood and lymphatic system disorders | Immune-mediated cytopenia | 6 | 107.79 | 45.82 | 253.58 | Y | 6.56 | 1.55 | 3.88 | Y |
| Blood and lymphatic system disorders | Granulomatous lymphadenitis | 4 | 22.03 | 8.15 | 59.54 | Y | 4.42 | 0.76 | 3.39 | Y |
| Blood and lymphatic system disorders | Lymphadenopathy mediastinal | 10 | 6.04 | 3.24 | 11.26 | Y | 2.58 | 1.17 | 2.92 | Y |
| Hepatobiliary disorders | Biliary fistula | 3 | 23.1 | 7.32 | 72.86 | Y | 4.49 | 0.35 | 3.29 | Y |
| Hepatobiliary disorders | Immune-mediated hepatitis | 177 | 131.33 | 111.92 | 154.1 | Y | 6.8 | 5.87 | 6.34 | Y |
| Hepatobiliary disorders | Granulomatous liver disease | 4 | 6.02 | 2.25 | 16.11 | Y | 2.58 | 0.29 | 2.88 | Y |
| Investigations | Thyroxine decreased | 6 | 16.4 | 7.3 | 36.83 | Y | 4.01 | 1.24 | 3.45 | Y |
| Investigations | Aspartate aminotransferase | 2 | 8.34 | 2.07 | 33.59 | N | 3.05 | -0.41 | 2.95 | N |
| Investigations | Blood lactate dehydrogenase abnormal | 4 | 11.7 | 4.36 | 31.41 | Y | 3.53 | 0.59 | 3.19 | Y |
| Renal and urinary disorders | Urinary tract obstruction | 10 | 5.23 | 2.81 | 9.73 | Y | 2.38 | 1.04 | 2.78 | Y |
| Renal and urinary disorders | Nephritis | 47 | 19.32 | 14.46 | 25.81 | Y | 4.24 | 3.36 | 4.2 | Y |
| Renal and urinary disorders | Autoimmune nephritis | 25 | 108.47 | 71.32 | 164.98 | Y | 6.57 | 3.76 | 4.96 | Y |
| Infections and infestations | Large intestine infection | 7 | 6.51 | 3.09 | 13.71 | Y | 2.69 | 0.92 | 2.97 | Y |
| Infections and infestations | Streptococcal urinary tract infection | 3 | 24.6 | 7.79 | 77.69 | Y | 4.58 | 0.35 | 3.3 | Y |
| Musculoskeletal and connective tissue disorders | Musculoskeletal toxicity | 3 | 18.55 | 5.9 | 58.33 | Y | 4.18 | 0.31 | 3.24 | Y |
| Musculoskeletal and connective tissue disorders | Autoimmune arthritis | 12 | 109.11 | 59.56 | 199.89 | Y | 6.58 | 2.67 | 4.38 | Y |
| Neoplasms benign, malignant and unspecified | Metastases to spleen | 3 | 10.34 | 3.31 | 32.3 | Y | 3.35 | 0.17 | 3.08 | Y |
| Neoplasms benign, malignant and unspecified | Neoplasm recurrence | 1 | 0.41 | 0.06 | 2.93 | N | -1.28 | -2.82 | 1.26 | N |
| Cardiac disorders | Pleuropericarditis | 3 | 7.65 | 2.45 | 23.85 | Y | 2.92 | 0.07 | 2.97 | Y |
| Ear and labyrinth disorders | Deafness bilateral | 9 | 7.47 | 3.87 | 14.41 | Y | 2.89 | 1.26 | 3.09 | Y |
| Metabolism and nutrition disorders | Cell death | 11 | 5.39 | 2.98 | 9.75 | Y | 2.42 | 1.14 | 2.81 | Y |
| Reproductive system and breast disorders | Spermatogenesis abnormal | 4 | 71.86 | 25.76 | 200.4 | Y | 6.04 | 0.88 | 3.59 | Y |
| Respiratory, thoracic and mediastinal disorders | Autoimmune lung disease | 7 | 114.82 | 51.84 | 254.33 | Y | 6.64 | 1.81 | 3.99 | Y |

Table 10 Sensitivity analysis stratified by reporting country (United States Subset)

| SOC | PT | Frequency | ROR | ROR_025_ | ROR_975_ | ROR_Signal | IC | IC_025_ | IC_975_ | IC_Signal |
| --- | --- | --- | --- | --- | --- | --- | --- | --- | --- | --- |
| Eye disorders | Iridocyclitis | 17 | 54.99 | 33.94 | 89.09 | Y | 5.74 | 3.08 | 4.46 | Y |
| Eye disorders | Iris adhesions | 1 | 25.37 | 3.53 | 182.52 | N | 4.65 | -1.12 | 3.01 | N |
| Eye disorders | Corneal perforation | 2 | 26.84 | 6.64 | 108.4 | N | 4.73 | -0.2 | 3.16 | N |
| Eye disorders | Choroidal effusion | 3 | 31.04 | 9.92 | 97.16 | Y | 4.93 | 0.41 | 3.32 | Y |
| Eye disorders | Autoimmune uveitis | 1 | 108.93 | 14.49 | 818.55 | N | 6.69 | -1.16 | 3.13 | N |
| Eye disorders | Autoimmune retinopathy | 1 | 80.51 | 10.87 | 596.21 | N | 6.27 | -1.14 | 3.1 | N |
| Nervous system disorders | Myelitis transverse | 3 | 9.18 | 2.95 | 28.56 | Y | 3.19 | 0.14 | 3.04 | Y |
| Nervous system disorders | Acute motor axonal neuropathy | 3 | 213.68 | 64.67 | 706.06 | Y | 7.58 | 0.44 | 3.51 | Y |
| Nervous system disorders | Miller Fisher syndrome | 2 | 84.17 | 20.4 | 347.25 | N | 6.33 | -0.17 | 3.27 | N |
| Nervous system disorders | Radiculopathy | 3 | 3.03 | 0.98 | 9.41 | N | 1.6 | -0.44 | 2.45 | N |
| Nervous system disorders | Peripheral sensorimotor neuropathy | 3 | 26.21 | 8.38 | 81.92 | Y | 4.69 | 0.38 | 3.3 | Y |
| Gastrointestinal disorders | Colonic fistula | 2 | 15.18 | 3.77 | 61.04 | N | 3.91 | -0.27 | 3.08 | N |
| Gastrointestinal disorders | Oral lichenoid reaction | 3 | 370.39 | 107.22 | 1279.53 | Y | 8.27 | 0.4 | 3.57 | Y |
| Gastrointestinal disorders | Gastrointestinal toxicity | 5 | 9.89 | 4.11 | 23.83 | Y | 3.3 | 0.81 | 3.17 | Y |
| Endocrine disorders | Immune-mediated thyroiditis | 20 | 579.27 | 350.53 | 957.27 | Y | 8.78 | 3.62 | 5.03 | Y |
| Endocrine disorders | Glucocorticoid deficiency | 1 | 47.48 | 6.52 | 345.62 | N | 5.53 | -1.12 | 3.06 | N |
| Blood and lymphatic system disorders | Immune-mediated cytopenia | 1 | 168.34 | 21.73 | 1303.99 | N | 7.27 | -1.2 | 3.18 | N |
| Hepatobiliary disorders | Biliary fistula | 1 | 84.17 | 11.34 | 624.49 | N | 6.33 | -1.14 | 3.11 | N |
| Hepatobiliary disorders | Immune-mediated hepatitis | 74 | 603.43 | 464.05 | 784.67 | Y | 8.83 | 5.64 | 6.38 | Y |
| Hepatobiliary disorders | Granulomatous liver disease | 1 | 8.73 | 1.22 | 62.3 | N | 3.12 | -1.21 | 2.89 | N |
| Investigations | Thyroxine decreased | 3 | 19.63 | 6.29 | 61.24 | Y | 4.28 | 0.34 | 3.25 | Y |
| Investigations | Aspartate aminotransferase | 1 | 30.86 | 4.28 | 222.71 | N | 4.92 | -1.12 | 3.03 | N |
| Investigations | Blood lactate dehydrogenase abnormal | 1 | 6.36 | 0.89 | 45.33 | N | 2.67 | -1.26 | 2.84 | N |
| Renal and urinary disorders | Urinary tract obstruction | 5 | 7.69 | 3.2 | 18.51 | Y | 2.94 | 0.68 | 3.04 | Y |
| Renal and urinary disorders | Nephritis | 29 | 60.09 | 41.5 | 87.01 | Y | 5.86 | 3.79 | 4.86 | Y |
| Renal and urinary disorders | Autoimmune nephritis | 4 | 137.19 | 49.68 | 378.84 | Y | 7 | 0.93 | 3.62 | Y |
| Infections and infestations | Large intestine infection | 2 | 4.69 | 1.17 | 18.8 | N | 2.23 | -0.6 | 2.74 | N |
| Infections and infestations | Streptococcal urinary tract infection | 1 | 19.09 | 2.66 | 136.91 | N | 4.24 | -1.14 | 2.99 | N |
| Musculoskeletal and connective tissue disorders | Autoimmune arthritis | 4 | 117.59 | 42.8 | 323.09 | Y | 6.79 | 0.93 | 3.61 | Y |
| Neoplasms benign, malignant and unspecified | Neoplasm recurrence | 11 | 15.56 | 8.59 | 28.17 | Y | 3.95 | 1.97 | 3.64 | Y |
| Ear and labyrinth disorders | Deafness bilateral | 1 | 3.2 | 0.45 | 22.74 | N | 1.68 | -1.44 | 2.65 | N |
| Reproductive system and breast disorders | Spermatogenesis abnormal | 4 | 569.86 | 185.79 | 1747.87 | Y | 8.77 | 0.85 | 3.77 | Y |

Table 11 Comparison of baseline characteristics between included and excluded cases in dose-severity analysis

| **Characteristic** | **Included**  N = 271^1^ | **Excluded**  N = 404^1^ | **p-value**^2^ |
| --- | --- | --- | --- |
| **Age_group** |  |  | 0.446 |
| <18 | 0 (0.0%) | 2 (0.8%) |  |
| ≥75 | 31 (12.5%) | 36 (13.6%) |  |
| 18-44 | 27 (10.9%) | 36 (13.6%) |  |
| 45-64 | 116 (46.8%) | 124 (46.8%) |  |
| 65-74 | 74 (29.8%) | 67 (25.3%) |  |
| Missing | 23 | 139 |  |
| **Gender** |  |  | 0.685 |
| Female | 103 (40.7%) | 114 (38.6%) |  |
| Male | 150 (59.3%) | 181 (61.4%) |  |
| Missing | 18 | 109 |  |
| **Cancer_type** |  |  | <0.001 |
| Bladder cancer | 2 (0.7%) | 2 (0.5%) |  |
| Colorectal cancer | 5 (1.8%) | 2 (0.5%) |  |
| Endometrial cancer | 0 (0.0%) | 2 (0.5%) |  |
| Gastric cancer | 1 (0.4%) | 0 (0.0%) |  |
| Hepatocellular carcinoma | 3 (1.1%) | 3 (0.7%) |  |
| Hodgkin lymphoma | 0 (0.0%) | 2 (0.5%) |  |
| Malignant melanoma | 167 (61.6%) | 220 (54.5%) |  |
| Non-small cell lung cancer | 16 (5.9%) | 28 (6.9%) |  |
| Other Cancers | 18 (6.6%) | 84 (20.8%) |  |
| Ovarian cancer | 1 (0.4%) | 1 (0.2%) |  |
| Pancreatic cancer | 0 (0.0%) | 1 (0.2%) |  |
| Pleural mesothelioma | 4 (1.5%) | 7 (1.7%) |  |
| Prostate cancer | 2 (0.7%) | 3 (0.7%) |  |
| Renal cell carcinoma | 48 (17.7%) | 45 (11.1%) |  |
| Small cell lung cancer | 2 (0.7%) | 4 (1.0%) |  |
| Squamous cell carcinoma | 2 (0.7%) | 0 (0.0%) |  |
| **Severity** |  |  | <0.001 |
| Fatal | 35 (12.9%) | 36 (8.9%) |  |
| Hospitalized | 118 (43.5%) | 126 (31.2%) |  |
| Life-threatening | 31 (11.4%) | 18 (4.5%) |  |
| Non-serious | 87 (32.1%) | 224 (55.4%) |  |
| ^1^n (%) | | | |
| ^2^Pearson's Chi-squared test | | | |

Table 12 Comparison of baseline characteristics between included and excluded cases in Sankey diagram analysis

| **Characteristic** | **Included**  N = 499^1^ | **Excluded**  N = 176^1^ | **p-value**^2^ |
| --- | --- | --- | --- |
| **Age_group** |  |  | 0.729 |
| <18 | 2 (0.4%) | 0 (0.0%) |  |
| ≥75 | 66 (13.2%) | 1 (7.1%) |  |
| 18-44 | 62 (12.4%) | 1 (7.1%) |  |
| 45-64 | 234 (46.9%) | 6 (42.9%) |  |
| 65-74 | 135 (27.1%) | 6 (42.9%) |  |
| Missing | 0 | 162 |  |
| **Gender** |  |  | 0.976 |
| Female | 197 (39.5%) | 20 (40.8%) |  |
| Male | 302 (60.5%) | 29 (59.2%) |  |
| Missing | 0 | 127 |  |
| **Cancer_type** |  |  | <0.001 |
| Bladder cancer | 4 (0.8%) | 0 (0.0%) |  |
| Colorectal cancer | 7 (1.4%) | 0 (0.0%) |  |
| Endometrial cancer | 2 (0.4%) | 0 (0.0%) |  |
| Gastric cancer | 1 (0.2%) | 0 (0.0%) |  |
| Hepatocellular carcinoma | 6 (1.2%) | 0 (0.0%) |  |
| Hodgkin lymphoma | 1 (0.2%) | 1 (0.6%) |  |
| Malignant melanoma | 317 (63.5%) | 70 (39.8%) |  |
| Non-small cell lung cancer | 29 (5.8%) | 15 (8.5%) |  |
| Other Cancers | 37 (7.4%) | 65 (36.9%) |  |
| Ovarian cancer | 2 (0.4%) | 0 (0.0%) |  |
| Pancreatic cancer | 1 (0.2%) | 0 (0.0%) |  |
| Pleural mesothelioma | 9 (1.8%) | 2 (1.1%) |  |
| Prostate cancer | 3 (0.6%) | 2 (1.1%) |  |
| Renal cell carcinoma | 75 (15.0%) | 18 (10.2%) |  |
| Small cell lung cancer | 3 (0.6%) | 3 (1.7%) |  |
| Squamous cell carcinoma | 2 (0.4%) | 0 (0.0%) |  |
| **Severity** |  |  | 0.001 |
| Fatal | 56 (11.2%) | 15 (8.5%) |  |
| Hospitalized | 187 (37.5%) | 57 (32.4%) |  |
| Life-threatening | 45 (9.0%) | 4 (2.3%) |  |
| Non-serious | 211 (42.3%) | 100 (56.8%) |  |
| ^1^n (%) | | | |
| ^2^Pearson's Chi-squared test | | | |

Table 13 Comparison of baseline characteristics between included and excluded cases in cancer-specific analysis

| **Characteristic** | **Included**  N = 171^1^ | **Excluded**  N = 504^1^ | **p-value**^2^ |
| --- | --- | --- | --- |
| **Age_group** |  |  | 0.206 |
| <18 | 0 (0.0%) | 2 (0.6%) |  |
| 18-44 | 14 (8.4%) | 49 (14.2%) |  |
| 45-64 | 79 (47.3%) | 161 (46.5%) |  |
| 65-74 | 47 (28.1%) | 94 (27.2%) |  |
| ≥75 | 27 (16.2%) | 40 (11.6%) |  |
| Missing | 4 | 158 |  |
| **Gender** |  |  | 0.305 |
| Female | 61 (36.1%) | 156 (41.2%) |  |
| Male | 108 (63.9%) | 223 (58.8%) |  |
| Missing | 2 | 125 |  |
| **Cancer_type** |  |  | <0.001 |
| Bladder cancer | 0 (0.0%) | 4 (0.8%) |  |
| Colorectal cancer | 0 (0.0%) | 7 (1.4%) |  |
| Endometrial cancer | 0 (0.0%) | 2 (0.4%) |  |
| Gastric cancer | 0 (0.0%) | 1 (0.2%) |  |
| Hepatocellular carcinoma | 0 (0.0%) | 6 (1.2%) |  |
| Hodgkin lymphoma | 0 (0.0%) | 2 (0.4%) |  |
| Malignant melanoma | 109 (63.7%) | 278 (55.2%) |  |
| Non-small cell lung cancer | 20 (11.7%) | 24 (4.8%) |  |
| Other Cancers | 0 (0.0%) | 102 (20.2%) |  |
| Ovarian cancer | 0 (0.0%) | 2 (0.4%) |  |
| Pancreatic cancer | 0 (0.0%) | 1 (0.2%) |  |
| Pleural mesothelioma | 0 (0.0%) | 11 (2.2%) |  |
| Prostate cancer | 0 (0.0%) | 5 (1.0%) |  |
| Renal cell carcinoma | 42 (24.6%) | 51 (10.1%) |  |
| Small cell lung cancer | 0 (0.0%) | 6 (1.2%) |  |
| Squamous cell carcinoma | 0 (0.0%) | 2 (0.4%) |  |
| **Severity** |  |  | <0.001 |
| Fatal | 26 (15.2%) | 45 (8.9%) |  |
| Hospitalized | 101 (59.1%) | 143 (28.4%) |  |
| Life-threatening | 18 (10.5%) | 31 (6.2%) |  |
| Non-serious | 26 (15.2%) | 285 (56.5%) |  |
| ^1^n (%) | | | |
| ^2^Pearson's Chi-squared test | | | |

2.2 Supplementary Figures

Figure 1 Missing-data flow diagram of integrated analysis


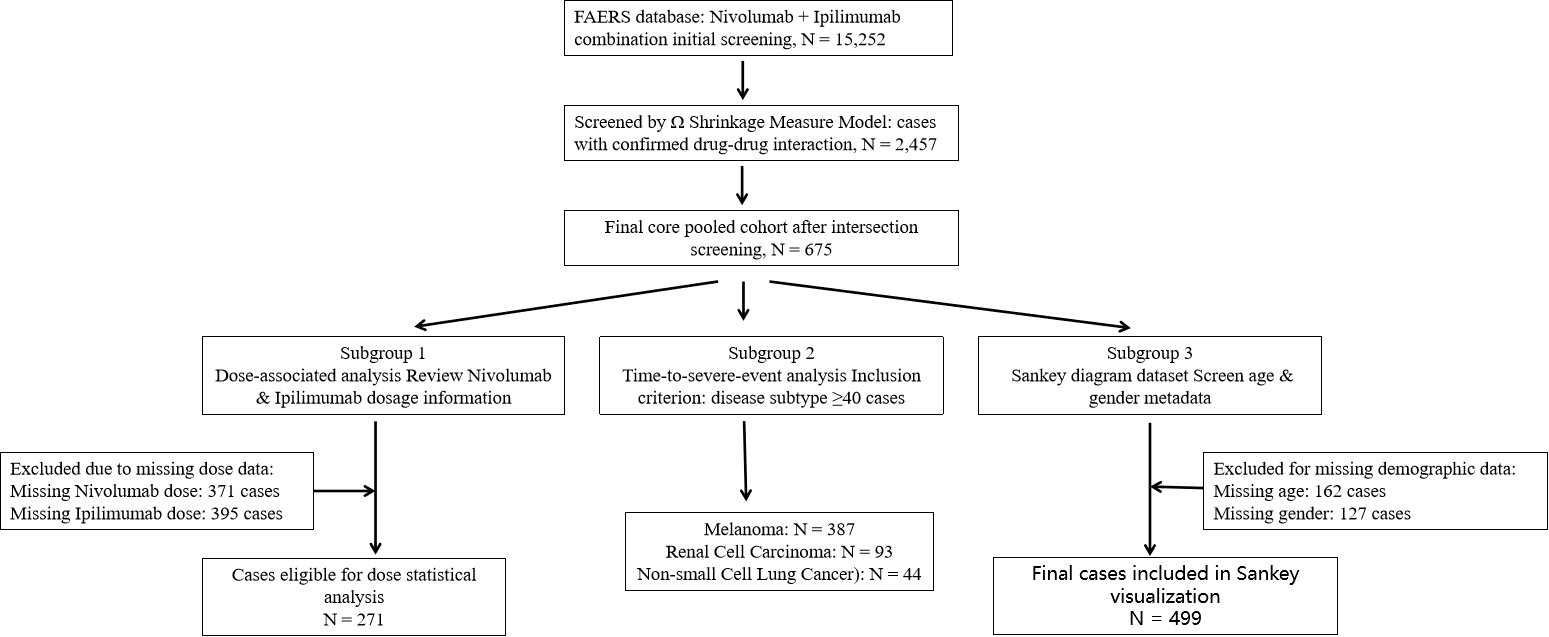

Supplement: Supplementary file 1 [file DataSheet1.docx]
